# Intravenous 5-fluoro-2′-deoxycytidine administered with tetrahydrouridine increases the proportion of p16-expressing circulating tumor cells in patients with advanced solid tumors

**DOI:** 10.1007/s00280-020-04073-5

**Published:** 2020-04-20

**Authors:** Geraldine O.’Sullivan Coyne, Lihua Wang, Jennifer Zlott, Lamin Juwara, Joseph M. Covey, Jan H. Beumer, Mihaela C. Cristea, Edward M. Newman, Stephen Koehler, Jorge J. Nieva, Agustin A. Garcia, David R. Gandara, Brandon Miller, Sonny Khin, Sarah B. Miller, Seth M. Steinberg, Larry Rubinstein, Ralph E. Parchment, Robert J. Kinders, Richard L. Piekarz, Shivaani Kummar, Alice P. Chen, James H. Doroshow

**Affiliations:** 1 Division of Cancer Treatment and Diagnosis, National Cancer Institute, 31 Center Drive, Bldg. 31 Room 3A-44, Bethesda, MD 20892 USA; 2 Clinical Pharmacodynamic Biomarkers Program, Applied/Developmental Research Directorate, Frederick National Laboratory for Cancer Research, Frederick, MD USA; 3 Clinical Monitoring Research Program, Clinical Research Directorate, Frederick National Laboratory for Cancer Research, Frederick, MD USA; 4 Department of Pharmaceutical Sciences, University of Pittsburgh School of Pharmacy, Pittsburgh, PA USA; 5 Department of Medical Oncology and Therapeutics Research, City of Hope National Medical Center, Duarte, CA USA; 6 City of Hope Medical Group, South Pasadena, CA USA; 7 University of Southern California Norris Comprehensive Cancer Center, Los Angeles, CA USA; 8 University of California Davis Cancer Center, Sacramento, CA USA; 9 Center for Cancer Research, National Cancer Institute, Bethesda, MD USA; 10 Present Address: Louisiana State University, New Orleans, LA 70112 USA

**Keywords:** DNMT1 inhibitors, Cancer epigenetics, Epigenetic modifying agents, Circulating tumor cells, p16

## Abstract

**Purpose:**

Following promising responses to the DNA methyltransferase (DNMT) inhibitor 5-fluoro-2′-deoxycytidine (FdCyd) combined with tetrahydrouridine (THU) in phase 1 testing, we initiated a non-randomized phase 2 study to assess response to this combination in patients with advanced solid tumor types for which tumor suppressor gene methylation is potentially prognostic. To obtain pharmacodynamic evidence for DNMT inhibition by FdCyd, we developed a novel method for detecting expression of tumor suppressor protein p16/INK4A in circulating tumor cells (CTCs).

**Methods:**

Patients in histology-specific strata (breast, head and neck [H&N], or non-small cell lung cancers [NSCLC] or urothelial transitional cell carcinoma) were administered FdCyd (100 mg/m^2^) and THU (350 mg/m^2^) intravenously 5 days/week for 2 weeks, in 28-day cycles, and progression-free survival (PFS) rate and objective response rate (ORR) were evaluated. Blood specimens were collected for CTC analysis.

**Results:**

Ninety-three eligible patients were enrolled (29 breast, 21 H&N, 25 NSCLC, and 18 urothelial). There were three partial responses. All strata were terminated early due to insufficient responses (H&N, NSCLC) or slow accrual (breast, urothelial). However, the preliminary 4-month PFS rate (42%) in the urothelial stratum exceeded the predefined goal—though the ORR (5.6%) did not. An increase in the proportion of p16-expressing cytokeratin-positive CTCs was detected in 69% of patients evaluable for clinical and CTC response, but was not significantly associated with clinical response.

**Conclusion:**

Further study of FdCyd + THU is potentially warranted in urothelial carcinoma but not NSCLC or breast or H&N cancer. Increase in the proportion of p16-expressing cytokeratin-positive CTCs is a pharmacodynamic marker of FdCyd target engagement.

**Electronic supplementary material:**

The online version of this article (10.1007/s00280-020-04073-5) contains supplementary material, which is available to authorized users.

## Introduction

Many malignancies are characterized by DNA methylation-mediated silencing of tumor suppressor gene expression. DNA methyltransferase (DNMT) enzymes catalyze the addition of a methyl group at the 5 position of cytosine residues found within CpG dinucleotide-rich islands throughout the genome, and frequent overexpression of DNMTs in tumor cells yields increased methylation of CpG islands within promoters and other regulatory regions [1]. This hypermethylation recruits proteins involved in heterochromatin formation, leading to transcriptional repression. Genes found in transcriptionally repressed regions within cancer cell nuclei include those encoding proteins involved in the regulation of cell cycle progression, DNA repair, apoptosis, and drug metabolism—such as the cyclin-dependent kinase (CDK) 4/6 inhibitor p16/INK4A (encoded by *CDKN2A*), von Hippel Lindau tumor suppressor gene (*VHL*), retinoblastoma 1 (*RB1*), and MutL protein homologue 1 (*MHL1*) [1, 2].

*CDKN2A* is one of the most frequently methylated genes across common cancer types and is often differentially silenced in primary tumors and tumor cell lines relative to non-malignant cells [2]. *CDKN2A* methylation and/or p16 expression has been shown to have prognostic value in non-small cell lung cancer (NSCLC), bladder cancer, and head and neck (H&N) cancers [3–6]. The potential of p16 as a pharmacodynamic (PD) biomarker for DNMT inhibitors has also been demonstrated, with treatment-induced increases in p16 expression observed in patient tumors from a phase 1 trial of decitabine in lung and esophageal cancers [7]. Thus, p16 expression represents a promising approach for monitoring the PD effects of DNMT inhibition.

Two DNA hypomethylating agents, 5-aza-2′deoxycytidine (decitabine) and 5-azacytidine (azacytidine), are FDA-approved for treatment of specific forms of acute myeloid leukemia, chronic myelomonocytic leukemia, and myelodysplastic syndromes—with response rates of approximately 50% across these diseases [8]. In contrast, monotherapy studies with demethylating agents in patients with advanced solid tumors have yielded only modest clinical activity and substantial toxicity, presumably due to cytotoxic nucleoside analog metabolites [9].

FdCyd, or 5-fluoro-2′-deoxycytidine, is a fluoropyrimidine nucleoside analog that, as has been demonstrated in vitro, is tri-phosphorylated and subsequently incorporated into DNA, where it covalently binds DNMT to inhibit DNA methylation [10, 11]. Unlike decitabine and azacytidine, FdCyd is stable in aqueous solution. However, like other cytidine analogs, FdCyd is rapidly metabolized in vitro and in humans and other animals by cytidine deaminase, forming the cytotoxic DNA replication inhibitor 5-fluoro-2′-deoxyuridine (FdUrd) [12–14]. Co-administration with the cytidine deaminase inhibitor tetrahydrouridine (THU) has been shown to increase in vivo FdCyd antitumor activity [14] and exposure [12, 13], attenuating levels of the cytotoxic FdUrd metabolite.

In our phase 1 study of FdCyd combined with THU, the combination was well tolerated and elicited a partial response (PR) that was sustained for 16 months in a patient with refractory breast cancer [15]. Therefore, we conducted a multicenter phase 2 study to determine the objective response rate (ORR) and progression-free survival (PFS) for FdCyd + THU in 4 strata—each specific to a cancer type for which there was clinical or preclinical evidence that tumor suppressor gene methylation may be associated with prognosis: breast [16, 17], head and neck [18, 19], and non-small cell lung [3, 5, 20] cancers and urothelial transitional cell carcinoma [6, 21, 22]. In addition to the main objectives of determining ORR and PFS, we also assessed toxicity, pharmacokinetics (PK), and PD responses to this regimen.

Given the extended timeframe of molecular response to epigenetic-modulating agents, we performed longitudinal PD assessments using liquid biopsies in this phase 2 study. Pharmacodynamic measurements in circulating tumor cells (CTCs) enabled PD response monitoring at multiple time points throughout treatment. FdCyd target engagement was assessed by measuring downstream expression of p16 in CTCs isolated from blood specimens using the FDA-cleared 4-channel CellSearch^®^ system, which utilizes EpCAM and CD146 capture beads [23]. Initially, we focused this analysis on epithelial-phenotype (cytokeratin-positive, putatively EpCAM-expressing) CTCs. However, during the course of the trial, new knowledge came to light regarding the biological relevance and potential prognostic value of mesenchymal- and mixed epithelial/mesenchymal (E/M)-phenotype CTCs in patients with metastatic disease [24–27]. Therefore, we developed and validated a novel 5-channel CellSearch^®^ assay to assess treatment-induced changes in p16 expression in the putative mixed E/M-phenotype (vimentin-positive, putatively EpCAM-expressing) CTC subpopulation for patients who enrolled during the final years of the study, after validation of this assay for E/M-phenotype CTCs had been completed. On this laboratory-specific platform, CTCs were identified as MUC1^+^/CD45^–^.

## Materials and methods

### Eligibility criteria

Patients age 18 years and older with histologically confirmed breast cancer, NSCLC, H&N cancer, or urothelial transitional cell carcinoma whose disease had progressed after at least one line of standard therapy were enrolled. Patients were required to have a Karnofsky performance status of ≥ 60% and adequate organ and marrow function, as defined by platelet count ≥ 100,000/μL, absolute neutrophil count ≥ 1500/μL, total bilirubin < 1.5 × the institutional upper limit of normal (ULN), alanine aminotransferase and/or aspartate aminotransferase ≤ 3 × ULN (or, for patients with liver metastases, ≤ 5 × ULN), and creatinine < 1.5 × ULN (or, for patients with levels > 1.5 × ULN, creatinine clearance of ≥ 60 mL/min). Patients were required to have completed any prior therapies ≥ 4 weeks prior to enrollment and must have recovered to eligibility levels for performance status and organ function following any prior toxicities.

### Trial design

This multicenter study (ClinicalTrials.gov identifier: NCT00978250) was conducted under an NCI-held investigational new drug application, with institutional review board approval at each participating site. This study was comprised of 4 strata based on tumor type: breast cancer, NSCLC, H&N cancer, and urothelial transitional cell carcinoma.

FdCyd and THU were supplied by the NCI Division of Cancer Treatment of Diagnosis. Patients were administered the recommended phase 2 doses and schedules: FdCyd (100 mg/m^2^/day) by 3-h intravenous infusion in 5% dextrose, and THU (350 mg/m^2^/day) in part as a bolus (20% of the daily dose), with the remaining co-administered with FdCyd over 3-h infusion on days 1–5 and 8–12 of each 28-day cycle. Patients maintained a study diary to note any side effects experienced or concurrent medications taken. The NCI Common Terminology Criteria for Adverse Events version 4.0 was used to grade adverse events. For drug-related toxicities ≥ grade 3, both study drugs were withheld until toxicities recovered to ≤ grade 2; upon re-initiation, the FdCyd dose was reduced per protocol depending on the degree of toxicity. Tumor response was assessed by radiography at baseline and every 2 cycles thereafter and evaluated per RECIST version 1.1 [28].

### Pharmacokinetics

See Supplementary Methods for information regarding pharmacokinetic analyses.

### Preclinical validation of a CellSearch^®^ assay for CTC p16 expression following FdCyd + THU treatment

Methodology and results concerning the development and preclinical validation of a CellSearch^®^ assay to quantitate the proportion of p16-expressing tumor cells are described in the Supplementary Methods, with data shown in Supplementary Fig. S1.

### CellSearch^®^ analysis of CTC p16 expression in patient blood specimens

#### Sample collection and processing

Blood specimens (7.5 mL each) were collected for CTC analysis at the following time points: prior to drug administration on cycle 1 day 1 (C1D1); on cycle 1 day 2 (C1D2; up to 24 h after end of infusion); on cycle 1 day 12 (C1D12); and on day 1 and day 12 of cycles 2, 4, and 6. Blood was collected into 10-mL CellSave Preservative Tubes (Menarini Silicon Biosystems). Tubes were inverted 8 times to distribute the anticoagulant and preservative and then stored at room temperature (for up to 96 h) until processing. Each blood sample was mixed with dilution buffer from the CellSearch^®^ Circulating Endothelial Cell (CEC) Kit (Menarini Silicon Biosystems) to a total volume of 14 mL, and then centrifuged and processed using the CellSearch^®^ platform. CTCs were assessed using an in-house-laboratory—developed test entailing dual capture of CTCs using CellSearch^®^ anti-EpCAM—and anti-MCAM (CD146)-coated beads, anti-CD45 antibodies to exclude PBMCs from the analysis, anti-pan-cytokeratin, and custom conjugated antibodies to detect p16, the mesenchymal marker vimentin (VIM), and the tumor marker mucin 1 (MUC1): AF 488-conjugated anti-p16 (clone EP435Y-129R, Abcam, Cambridge, UK), phycoerythrin (PE)-conjugated anti-vimentin (clone V9, Santa Cruz Biotechnology, Dallas, TX), and PerCP-Cy5.5-conjugated anti-MUC1 (clone number E29, Santa Cruz Biotechnology). The E29 anti-MUC1 monoclonal antibody binds to a portion of the MUC1 tandem repeat sequence in a glycosylation-independent fashion [29, 30]. This method captures circulating cells that are either EpCAM- or CD146-positive, identifies captured cells that express MUC1, and can identify high numbers of PD biomarker/MUC1-double-positive cells. Importantly, our pilot analysis of 18 healthy donor blood specimens (6 donors, three 7.5-mL specimens per donor) found no CK^+^/MUC1^+^/ DAPI^+^/CD45^−^ or VIM^+^/MUC1^+^/DAPI^+^/CD45^−^ cells in these specimens, demonstrating the specificity of this assay for circulating tumor cells.

#### CTC classification

Images captured by the 5-Color System in CellTracks^®^ Analyzer II contain objects fulfilling predetermined criteria and are automatically presented in gallery format. Final classification of cells was performed independently by two operators. Cells were classified as CTCs when morphologic features and staining patterns were consistent with that of epithelial-phenotype circulating tumor cells (i.e., CK-positive, DAPI-positive, CD45-negative, and tumor marker [MUC1]-positive) or putative mixed E/M-phenotype circulating tumor cells (vimentin-positive, DAPI-positive, CD45-negative, and tumor marker-positive). CTCs were required to have a minimum size of at least 4 μm, though CTCs presented with substantial heterogeneity in size and morphology.

#### Pharmacodynamic analyses in cytokeratin- or vimentin-positive CTCs

Serial baseline samples from individual patients (collected on C1D1 and C1D2) were used to determine variability in baseline levels of CTCs and changes in vimentin-, cytokeratin-, and/or p16-positive CTCs in response to treatment. Patients were prospectively designated as assessable for vimentin-positive (VIM^+^) or cytokeratin-positive (CK^+^) CTC p16 response if at least one of the baseline patient blood specimens (i.e., C1D1 and/or C1D2) contained ≥ 6 CTCs, and at least one post-treatment patient blood specimen (i.e., specimens collected C1D12 or later) contained ≥ 6 CTCs. Six CTCs was selected as the cut-off to minimize errors in p16 expression classification due to small sample size. For examining changes in p16 prevalence, both C1D1 and C1D2 samples were prospectively considered baseline specimens to obtain more accurate measurements, given the known variability in baseline CTC count; for patients with specimens from both time points, we took a stringent approach by assigning the specimen with the highest frequency of CTC p16 expression as the baseline specimen for assessing post-treatment p16 positivity. Patients were designated as having undergone an increase in the proportion of p16-expressing CTCs according to the following prospectively defined criteria: if the percentage of p16-positive CTCs increased by at least threefold (or from 0 to ≥ 2 p16-positive CTCs in specimens containing ≥ 6 CTCs each) in one or more post-treatment specimens relative to baseline.

### Statistical analysis

The study was designed to include co-primary endpoints of ORR and PFS, with a successful outcome in either parameter designating the regimen as worthy of further testing. The study design was intended to discriminate between response rates of 20% versus 5% or 4-month (or, for the breast stratum, 6-month) PFS probabilities of 50% vs. 25% (corresponding to median PFS of 4 vs. 2 months, or, for the breast cancer stratum, 6 vs. 3 months); see Supplementary Materials for additional details. Based on survival and response metrics from prior studies (see Supplementary Materials), FdCyd combined with THU was designated as worthy of further testing in NSCLC, urothelial, or H&N cancer if ≥ 6 objective responses (≥ 13%), or ≥ 18 instances of 4-month PFS (≥ 40%), were observed among 45 enrolled patients; for the breast stratum, the regimen was considered worthy of further testing if ≥ 5 objective responses (≥ 14%), or ≥ 15 instances of 6-month PFS (≥ 43%), were observed among 35 enrolled patients. Design of the NSCLC, urothelial, and H&N strata included provisions for the early termination due to insufficient antitumor activity: if no more than one objective response (≤ 5%), and no more than six instances of 4-month PFS (≤ 30%), were observed among the initial 20 patients.

ORR was calculated by dividing the number of responses by the number of eligible patients, per RECIST version 1.1 [28]. PFS was calculated from the start of therapy until date of progression or death without progression, using the Kaplan–Meier method. Patients who were taken off study for refusal of further treatment, intercurrent illness, PI discretion, or other were censored at the date therapy ended.

A log-rank test was used to assess associations between baseline CK^+^ CTC count and PFS. A Fisher’s exact test was used to compare the clinical response rate (PR + stable disease [SD], vs. progressive disease [PD]) between patients with versus those without an increase in the proportion of p16-expressing CK^+^ CTCs; patients with high frequencies of CTCs positive for p16 (≥ 15%) at baseline were omitted from the analysis. All *p* values are two-tailed.

## Results

### Patient demographics

From September 2009 through December 2017, 95 patients were enrolled on this study; 93 were eligible, including 29 breast, 25 NSCLC, 21 H&N, and 18 urothelial cancer patients (Table 1 and Supplementary Fig. S2). The median patient age was 60 years (range 30–84 years). The patient population was heavily pre-treated, with a median of 6 prior therapies (median by stratum: breast, 10; NSCLC, 5; H&N, 4; urothelial, 3.5).

**Table 1:** Patient characteristics

Characteristics	Number of patients
Number of patients enrolled/eligible/evaluable for response	95/93/83
Median age, years (range)	60 (30–84)
Karnofsky performance status (%)	
100	12
90	28
80	35
70	16
60	3
Diagnosis (eligible/evaluable for response)	
Breast	29/28
NSCLC	25/24
Head and neck	21/16
Urothelial	18/15
Median number of prior therapies (range)	6 (1–25)

### Toxicity

Intravenous FdCyd combined with THU was well tolerated. As observed in the phase 1 study of this combination [15], the most commonly occurring drug-related grade 3/4 adverse events were hematologic toxicities, with gastrointestinal toxicities also prevalent (Supplementary Table S1).

### Efficacy

**Fig. 1:**
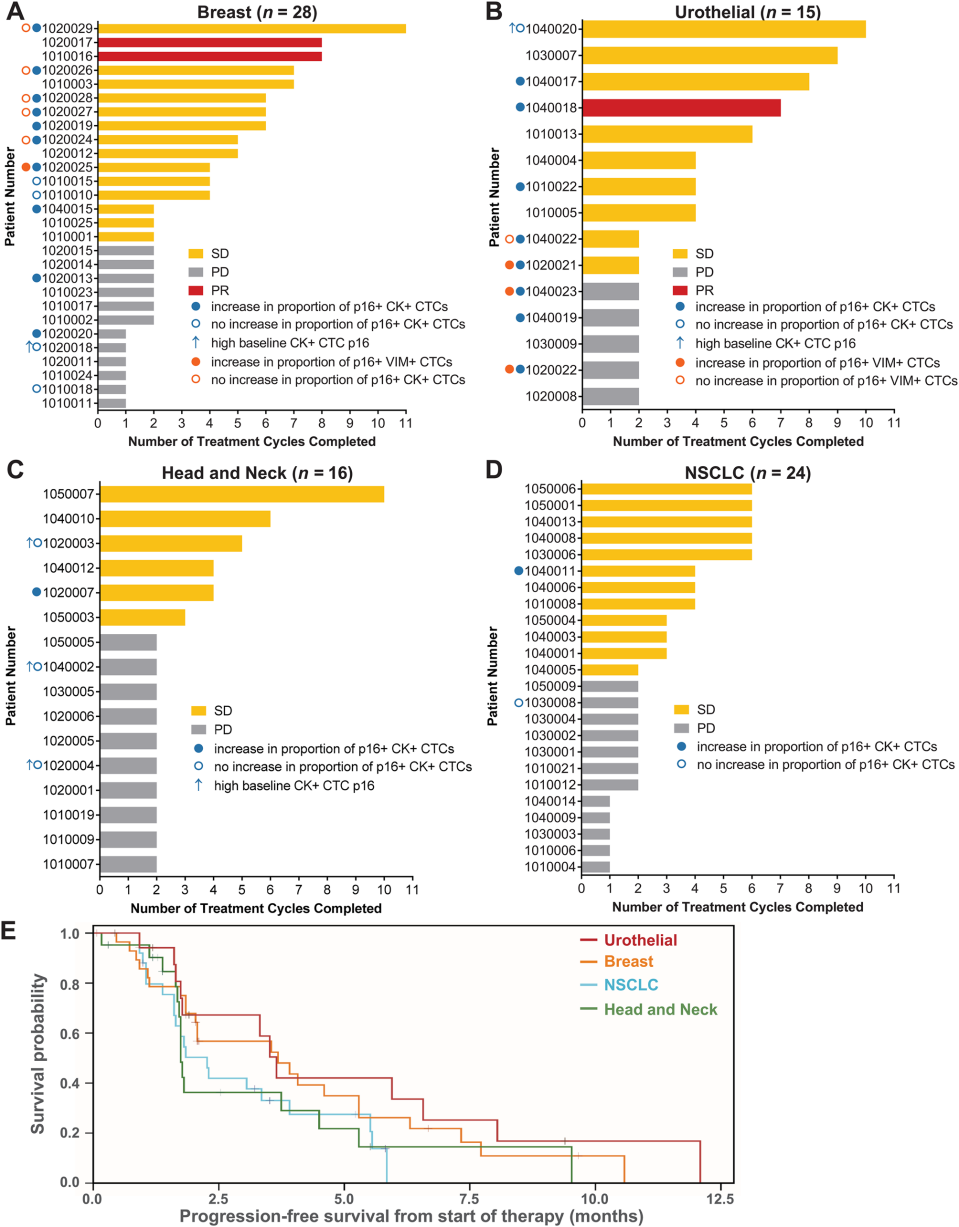
Clinical response and progression-free survival following FdCyd + THU therapy, by tumor type. **a**–**d** The number of cycles of treatment completed is shown for each patient evaluable for objective response, and patients for each tumor type stratum are shown (**a** breast; **b** urothelial; **c** head and neck; **d** NSCLC) along with the best response to therapy (partial response, red; stable disease, yellow; progressive disease, gray). Patients who did or did not exhibit an increase in the proportion of p16-expressing CTCs are indicated by filled or open circles, respectively, with orange circles indicating VIM^+^ CTCs and blue indicating CK^+^ CTCs; patients with high baseline p16 expression in CK^+^ CTCs are indicated by blue arrows. Patients with no circle shown were not evaluable for CTC p16 response. **e** Kaplan–Meier progression-free survival curves are shown for each stratum and include data for all 93 enrolled patients

Of the 93 patients, 83 were evaluable for objective response. Of the 10 patients who went off study prior to response assessment, 5 refused further treatment, 3 experienced toxicity, 1 experienced intercurrent illness, and 1 died of cardiac arrest possibly due to FdCyd (Supplementary Fig. S2). Best response and number of treatment cycles completed for each patient evaluable for objective response are shown in Fig. 1, along with Kaplan–Meier curves denoting PFS for all 93 patients across the 4 strata. Objective response rates were calculated based on the number of eligible patients, per current RECIST guidelines [28]. Three patients (1 urothelial, 2 breast) experienced a PR, for an overall objective response rate of 3.2%; ORRs for individual strata were 5.6%, 6.9%, 0%, and 0% for urothelial, breast, H&N, and NSCLC, respectively (Table 2 and Fig. 1). The median PFS for all strata combined was 3.1 months (95% confidence interval [CI] 1.8–3.7 months); median PFS values (and 95% CI) for individual strata were 3.6 (1.7–8.0), 3.7 (1.8–5.3), 1.7 (1.7–4.5), and 2.3 (1.6–3.9) months for urothelial, breast, H&N, and NSCLC, respectively. Four-month PFS probabilities (and 95% CI) for the urothelial, H&N, and NSCLC strata were 42.0 (16.2–66.1), 29.0 (10.0–51.5), and 27.5 (11.2–46.6), respectively, while the 6-month PFS probability for the breast stratum was 26.2 (95% CI 10.9–44.4) (Table 2). Based on response rate and PFS data, and per predefined early termination rules, the NSCLC and H&N strata were closed early due to insufficient antitumor activity.

**Table 2:** Objective response rates and progression-free survival by stratum

Stratum	ORR, % (95% CI)	Median PFS, months (95% CI)	4- or 6-month^a^ PFS probability, % (95% CI)
Overall (all 4 strata)	3.2 (0.7–9.1)	3.1 (1.8–3.7)	
Breast	6.9 (0.8–22.8)	3.7 (1.8–5.3)	26.2 (10.9–44.4)
Urothelial	5.6 (0.1–27.3)	3.6 (1.7–8.0)	42.0 (16.2–66.1)
Head and neck	0.0 (0–16.1)	1.7 (1.7–4.5)	29.0 (10.0–51.5)
NSCLC	0.0 (0.0–13.7)	2.3 (1.6–3.9)	27.5 (11.2–46.6)

ORR was calculated by dividing the number of responses by the total number of eligible patients

^a^Per protocol, 6-month PFS was used for analysis of the breast stratum; 4-month PFS was used for all other strata

The breast and urothelial strata were ultimately closed due to insufficient accrual (Table 1); this precluded comprehensive statistical comparisons of response and PFS probabilities relative to predefined thresholds specified in the protocol, which were based on enrollment of 35 and 45 evaluable patients for the breast and urothelial strata, respectively. Though accrual goals were not met for these strata, preliminary results suggest that further testing of FdCyd + THU in urothelial transitional cell carcinoma may be warranted, given the 4-month PFS probability of 42% (versus the predefined target probability of ≥ 40%) in the first 18 urothelial patients; however, the ORR of 5.6% did not meet the predefined target rate of ≥ 13% (Tables 1 and 2). Preliminary results for the breast stratum do not support further testing of FdCyd + THU in this disease (6.9% ORR and 26.2% 6-month PFS probability in the first 29 patients, versus predefined target values of ≥ 14% and ≥ 43%, respectively).

### Pharmacokinetic analysis

Plasma pharmacokinetic parameters were assessed for a subset of patients as described in the Supplementary Materials, and the resulting values (Supplementary Table S2 and Supplementary Fig. 3) were similar to those previously reported for IV administration of the same doses in the prior phase 1 study [15].

### Pharmacodynamic analysis

#### Cytokeratin-positive CTC enumeration

Though the presence of CTCs in baseline blood specimens is known to be of prognostic value in various indications [31–37], we found no significant association between pre-treatment (C1D1) cytokeratin-positive (CK^+^) CTC count and PFS amongst patients with pre-treatment CTC specimens (Supplementary Fig. S4a, b). Furthermore, median post-treatment changes in CK^+^ CTC counts did not appear to be appreciably different across categories of best response to therapy (PR, SD, or PD), though interpatient heterogeneity in CTC number was high (Supplementary Fig. S4c), as has been observed previously [31, 34, 38].

#### Cytokeratin-positive CTC p16 expression

Of the 83 patients evaluable for clinical response, 29 (35%) were also evaluable for changes in the proportion of p16-expressing CK^+^ CTCs according to our prospectively defined criteria (see Materials and Methods). The majority of patients who were not evaluable for p16 CTC response (87%; 47/54) did not have sufficient numbers of CTCs present at baseline and/or any post-treatment time point, while the remaining 13% (7/54) were lacking baseline and/or post-treatment blood specimens for CTC analysis.

Blood specimens for baseline p16 CTC analysis were collected on both C1D1 and C1D2 to address the known intra-patient variability in baseline CTC count and to account for drug-induced CTC showering that can occur in some patients [23, 38]. DNMT inhibitors such as FdCyd require an extended timeframe for activity, given that transition through S phase of the cell cycle is needed to enable inhibitor incorporation into DNA, and human tumors exhibit relatively slow progression through the cell cycle—resulting in tumor volume doubling times in the range of weeks to months [39]. In addition, prior experiments have shown that even in vitro, DNMT inhibitor-induced p16 protein expression in tumor cells occurs only after a 24–48-h lag period following treatment [40], and our EJ6 cell culture data show that appreciable upregulation of p16 protein expression (detected by Western blot) occurs beginning at 2 weeks after a single application of FdCyd + THU (Supplementary Figure S1a). Because of this extended timeframe for FdCyd-induced epigenetic effects, differences in CTC p16 expression on C1D1 vs. C1D2 are assumed to reflect baseline heterogeneity in p16 expression rather than pharmacodynamic effects. We took a conservative approach in defining treatment-induced increases in the proportion of p16-expressing CTCs, using the higher of the 2 C1D1 and C1D2 values as the baseline value. For the majority of patients evaluable for p16 response (51.7%; 15 of 29), the percent of CK^+^ CTCs expressing p16 was identical for the C1D1 and C1D2 specimens; 6 patients (20.7%) did not have an evaluable CTC blood specimen for 1 of the 2 baseline time points, and of the remaining 8 patients (27.6%), the median difference in % CTCs expressing p16 between the 2 time points was 1% (range − 1 to 33%).

**Fig. 2:**
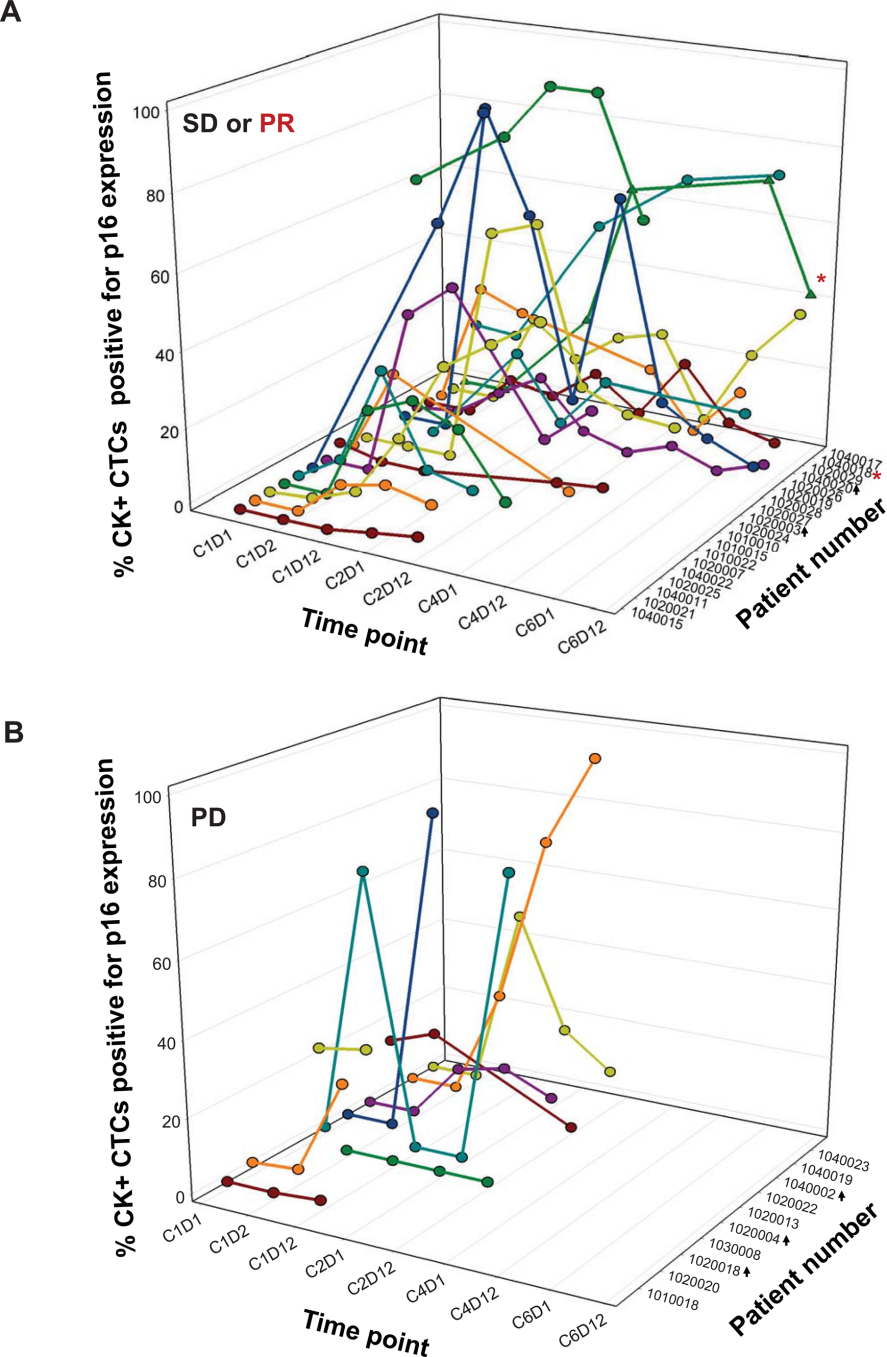
FdCyd + THU treatment increases the proportion of p16-expressing cytokeratin-positive circulating tumor cells. The percentages of CK^+^ CTCs also positive for p16 expression are shown at each time point (cycle, day) for all patients evaluable for both clinical response and CTC p16 expression, including patients with a best response of **a** stable disease (SD) or partial response (PR) or **b** progressive disease (PD). Each set of identically colored symbols and connecting lines represents data from a single patient. The patient who experienced a PR is indicated by a red asterisk (with data points shown as triangles), while patients with high baseline (C1D1 and/or C1D2 values ≥ 15%) p16 expression are denoted by black arrows. Within each graph, patients are ordered by increasing number of treatment cycles completed, from front to back

In selecting a prospective cut-off to define an increase in the proportion of p16-expressing CTCs, we used a ≥ threefold increase in the percentage of p16-expressing CTCs at any post-treatment time point, compared to baseline specimens (C1D1 or C1D2, as described above). For patients with no p16-expressing CTCs at baseline, an increase to ≥ 2 CTCs (in a specimen containing ≥ 6 CTCs) was used to define an increase in the proportion of p16-expressing CTCs. A treatment-induced increase in the proportion of p16-expressing CK^+^ CTCs was observed in 20 of 29 patients (69%) evaluable for both clinical response and changes in p16 expression (Table 3, Figs. 1 and 2). An increase in the proportion of p16-expressing CK^+^ CTCs was observed at the earliest non-baseline time point (C1D12) for most patients (Fig. 2).

**Table 3:** Patient response and therapy-associated increases in CK^+^ CTC p16 positivity

	PD	SD	PR
All patients evaluable for clinical response and CTC p16 expression changes (*n* = 29)			
No. pts with increase in proportion of p16-expressing CTCs	5	14	1
No. pts with no increase in proportion of p16-expressing CTCs	5	4	0
Patients with high baseline p16 removed (*n* = 24)			
No. pts with increase in proportion of p16-expressing CTCs	5	14	1
No. pts with no increase in proportion of p16-expressing CTCs	2	2	0

Among the 24 patients with low baseline p16 expression, no significant difference in the rate of PR + SD was observed for patients with an increase in the proportion of p16-expressing CK^+^ CTCs vs. those without (*p* = 0.55)

Of the 9 patients for whom no increase in the proportion of p16-expressing CK^+^ CTCs was observed at any time point, 5 had a high proportion (≥ 15%) of p16-positive CK^+^ cells at baseline (Table 3, Figs. 1 and 2), and these patients were omitted in the statistical analysis of associations between clinical response and treatment-induced increases in the proportion of p16-expressing CK^+^ CTCs; the other 4 patients with no increase in the proportion of p16-expressing CK^+^ CTCs had low baseline p16 expression (< 15%) and were included in the analysis of p16 expression changes and clinical response. In addition, one patient with a PR in the breast stratum (patient 1,020,017) and one patient with extended stable disease in the H&N stratum (patient 1,050,007) were classified as not evaluable for p16 CTC response, because they did not have a sufficient number of CTCs at baseline; however, these patients had evaluable numbers of CTCs at multiple later time points, and an increase in the proportion of p16-expressing CK^+^ CTCs was observed at these later time points (Figs. 1 and 2).

Of the 24 patients with low baseline p16 expression in CK^+^ CTCs, an increase in the proportion of p16-expressing CK^+^ CTCs was observed in 2 of 2 (100%) patients with a best response of PR, 13 of 15 (87%) patients with SD, and 5 of 7 (71%) of patients with PD (Table 3); however, the presence of just 4 patients with no increase in the proportion of p16-expressing CK^+^ CTCs limits the statistical power for examining associations between response and p16 expression. When comparing patients with a treatment-induced increase in the proportion of p16-expressing CK^+^ CTCs vs. those without, the resulting differences in PR + SD rates (75% vs. 50%) were not significant (*p* = 0.55). Therefore, although the clinical response rates for the two groups suggest that there may be modest prognostic value to an increased proportion of p16-expressing CK^+^ CTCs, the sample size is too small to achieve statistical significance. Likewise, sample sizes were too small to adequately evaluate histologic stratum-specific differences in CK^+^ CTC p16 response; however, 3 of 5 patients with high baseline p16 expression were from the H&N stratum (Fig. 1c), consistent with the high prevalence of p16 expression in tumors from H&N patients infected with human papilloma virus (HPV) [41].

#### Characterization of vimentin-positive CTCs

While CTC analyses have typically relied upon selection of epithelial-phenotype CTCs via EpCAM, CK, or other epithelial markers, mesenchymal- and mixed epithelial/mesenchymal-phenotype CTCs are, a priori, more consistent with the characteristics of metastatic tumor cells, including enhanced motility, invasiveness, and self-renewal [42]; indeed, data from several recent studies across multiple tumor types have suggested that mesenchymal- or mixed E/M-phenotype CTCs may be of greater prognostic and/or predictive value than their epithelial-phenotype counterparts [24–27]. Following the emergence of this information regarding the potential predictive value of mesenchymal- or mixed E/M-phenotype CTCs, we developed and validated a novel custom CellSearch^®^ assay to assess p16 expression in the putative mixed E/M-phenotype subpopulation. Because assay validation was completed during the late stages of this trial, we performed a pilot analysis of putative mixed E/M-phenotype CTCs in 13 of the final patients enrolled, employing MUC1 as a tumor marker as has been done in several prior CTC studies [43–45], and enumerating and measuring p16 expression in VIM^+^, MUC1^+^, DAPI^+^, and CD45^–^ cells from peripheral blood specimens. To assess all 5 markers, we implemented and validated a novel 5-color CellSearch^®^ assay (Supplementary Fig. S5a), which was made possible by a technological upgrade of the CellSearch^®^ system to add a fifth fluorescence channel. We established concordance between the 4- and 5-channel CellSearch^®^ systems for CTC enumeration and PD response evaluation (Supplementary Fig. S5b–e).

In general, there were substantially more VIM^+^/MUC1^+^/DAPI^+^/CD45^–^ cells (hereafter referred to as “VIM^+^ CTCs”) compared to CK^+^/MUC1^+^/DAPI^+^/CD45^–^ cells (“CK^+^ CTCs”) across all patients and time points (Supplementary Fig. S6a); the average ratio of VIM^+^:CK^+^ CTCs was 13.8 (median: 4.7; minimum: 0; maximum: 200.2). For several patients, the number of VIM^+^ CTCs appeared to increase in the cycle prior to their progression on FdCyd + THU, though the small sample size precludes adequately powered statistical analyses (Supplementary Fig. S6a).

**Fig. 3:**
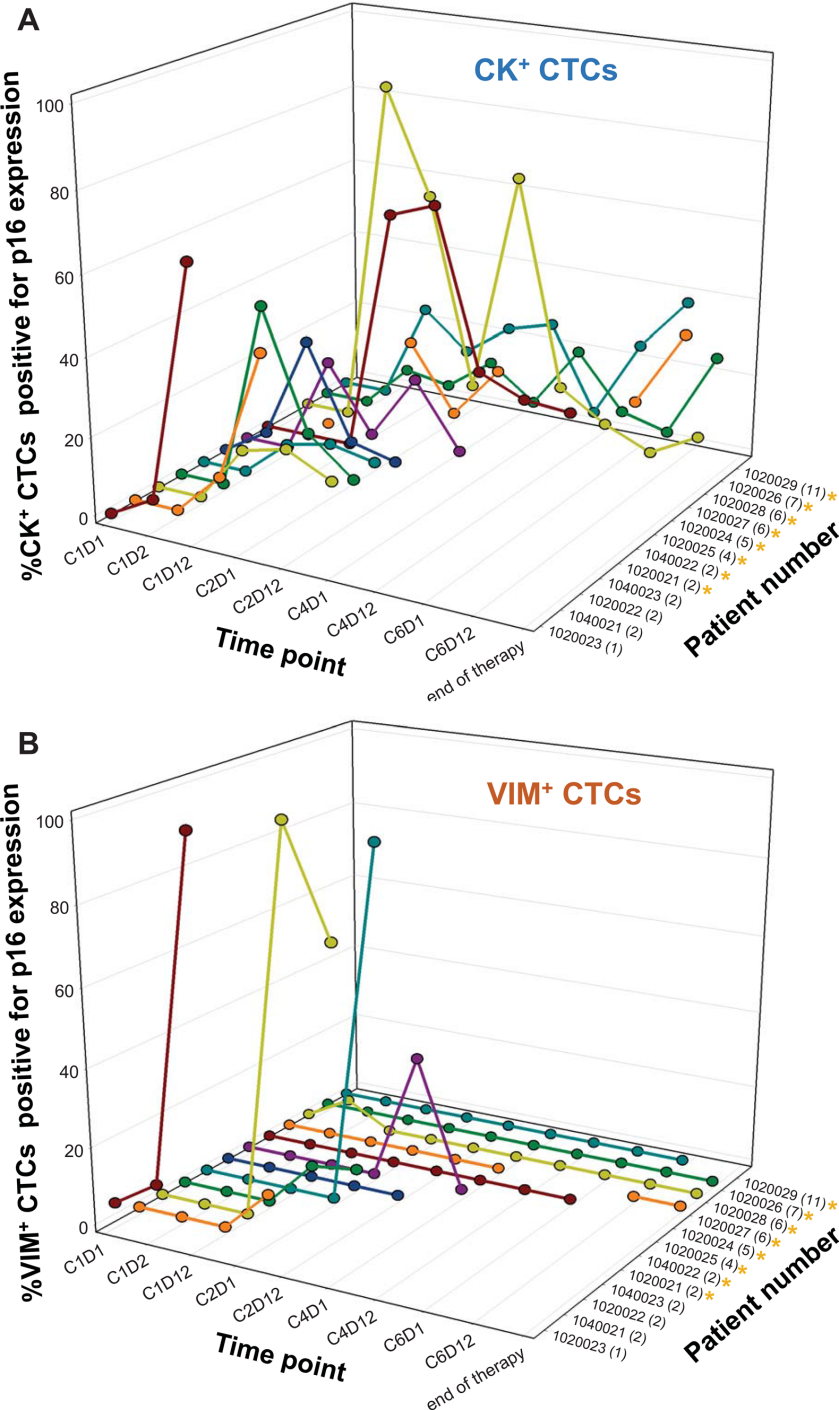
P16 expression in cytokeratin-positive versus vimentin-positive CTCs. Percentages of p16-positive CTCs of **a** epithelial phenotype (CK^+^) or putative epithelial/mesenchymal mixed phenotype (VIM^+^) are shown at each time point for each of 12 patients with CTC specimens evaluable for both CK^+^ and VIM^+^ CTC p16 response. Each set of colored symbols and connecting lines represents data from a single patient; order/coloring of patients is the same in (**a**, **b**). The number of cycles of therapy completed is noted in parentheses next to each patient number along the *x* axis. Gold asterisks denote patients with a best response of stable disease

We also examined p16 expression in VIM^+^ CTCs, using the criteria described for CK^+^ CTCs, and compared p16 expression patterns in VIM^+^ vs. CK^+^ CTCs. Twelve of thirteen patients were assessable for both CK^+^ and VIM^+^ CTC p16 expression changes, indicating that our laboratory-developed test—even with its requirement for tumor marker positivity—detected adequate CK^+^ CTC numbers (≥ 6) in a higher proportion of patients compared to the 4-channel CellSearch^®^ assay. Of these 12 patients, 6 (50%) did not show an increase in the proportion of p16-expressing VIM^+^ CTCs at any post-treatment time point; in contrast, an increase in the proportion of p16-expressing CK^+^ CTCs was observed for all 12 patients (Supplementary Fig. S6b). In addition, increases in the proportion of p16-expressing VIM^+^ CTCs, when they did occur, were slightly delayed relative to such changes in CK^+^ CTCs (Supplementary Fig. S6b). Increases in the proportion of p16-expressing VIM^+^ CTCs did not seem to be indicative of response to therapy, as some of the longest durations of stable disease in breast cancer patients occurred in patients for which no increase in the proportion of p16-expressing VIM^+^ CTCs was observed, and all 4 response-assessable urothelial patients with VIM^+^ CTC data were on study for just 2 cycles of treatment, regardless of VIM^+^ CTC p16 expression status (Figs. 1 and 3).

## Discussion

Our efficacy results suggest that further testing of the FdCyd + THU combination is potentially warranted only in patients with urothelial transitional cell carcinoma, but not in NSCLC, H&N cancer, or breast cancer. Lack of sufficient antitumor activity in the NSCLC and H&N strata warranted early termination of both, while preliminary results for the 29 patients in the breast stratum also indicated insufficient activity for further analysis. For the 18-patient urothelial stratum, the low ORR indicated insufficient activity, but the 4-month PFS probability essentially met the predefined cut-off selected to designate promising activity (though the cut-off was designed assuming accrual of 45 evaluable patients). Future studies of FdCyd + THU in a sufficient number of urothelial transitional cell carcinoma patients could enable more precise determination of the response rate and PFS; however, given the recent success of immune checkpoint inhibitors in this disease, further testing of FdCyd + THU should focus on a checkpoint blockade-refractory or -ineligible patient population. One limitation of the current study that may have impacted patient accrual and retention was the intravenous administration schedule, which required a total of ten 3-h infusions per month; this issue has been addressed by the development of an oral FdCyd formulation [13], and a phase 1 multicenter study of oral FdCyd + oral THU (NCT01534598) is ongoing [46].

While the multifaceted biological effects of DNMT inhibition complicate determination of the precise molecular basis for differences in FdCyd activity across strata, data from prior mechanistic studies may help to explain our results. Modest antitumor activity in our breast cancer patients may have been due to the varying relationship between DNA methylation and prognosis that has been observed across breast cancer subtypes [47, 48]; for example, DNA hypomethylation, rather than hypermethylation, is associated with poor survival in triple-negative breast cancer [47]. In addition, p16 overexpression, rather than loss, has been shown to correlate with hormone receptor negativity and poor outcome in some breast cancer patients [49, 50]. For NSCLC and H&N cancer, heterogeneity in the gene expression patterns driving malignancy may account for the lack of FdCyd + THU activity. For example, the prevalence of tumor p16 expression in HPV-positive H&N patients suggests that p16 silencing is not a driver of disease in these patients; in addition, a genome-wide DNA methylation analysis of H&N squamous cell carcinomas found substantial tumor DNA hypomethylation associated with HPV positivity, including hypomethylation of binding sites for the oncogenic transcription factor c-MYC [51]. Therefore, DNMT inhibitor-induced hypomethylation may not be a viable mechanism to control tumor growth in HPV-positive H&N cancers. In NSCLC, histologic subtype variations in the hypermethylation of p16 and other tumor suppressor genes [52] may account for lack of FdCyd clinical activity. We employed the CellSearch^®^ system for our primary correlative objective of monitoring pharmacodynamic responses to FdCyd + THU, though we also used this platform to assess the prognostic value of baseline CTC number based on results from the previous studies, showing that high baseline CTC counts are associated with poor survival [31–37] and, in some cases, with high tumor burden specifically [37, 53, 54]. Unlike these prior studies demonstrating the prognostic value of baseline CTC count, we found no association between pre-treatment CK^+^ CTC number and PFS, which may be due in part to the smaller sample size and more advanced disease status of patients in this study, particularly given the distribution of these patients across 4 different tumor types; prior studies demonstrating a prognostic value for baseline CTC count have largely focused on a single indication and various disease stages therein.

For pharmacodynamic analyses of CTCs, p16 was selected as a PD biomarker based on extensive prior literature, as well as our preclinical validation of antibody specificity and FdCyd-induced p16 expression changes. Use of the CellSearch^®^ platform for CTC pharmacodynamic analyses has several caveats, including the relatively low numbers of isolated EpCAM- or CK-expressing cells [23]. Indeed, we were able to identify many more VIM^+^ CTCs relative to CK^+^ CTCs, and this technological feasibility, combined with the greater potential biological relevance of mesenchymal or mixed E/M-phenotype CTCs in patients with metastatic disease, suggests that analysis of this CTC population may enable generation of more statistically robust and informative data in future studies. The CellSearch^®^ platform is also limited by the availability of just 5 channels for analysis (and only 4 channels at the time of study initiation), rendering full exploration of biological processes difficult; for example, the limited number of channels precluded measuring vimentin and CK simultaneously, preventing thorough analysis of mixed epithelial/mesenchymal phenotypes, as has been performed in the other studies [24–27].

We detected treatment-induced increases in the proportion of p16-expressing cytokeratin-positive CTCs for the majority (69%) of patients evaluable for both clinical response and CTC p16 expression changes, including in all such patients with low baseline p16 expression and a best response of PR or prolonged SD; however, an increase in the proportion of p16-expressing CK^+^ CTCs was not significantly associated with clinical response. Considering the relatively small sample size and limited number of responses, as well as the complexity of proposed mechanisms of action for cytidine analogs via general CpG island demethylation, this was not a surprising finding. One potential factor contributing to the small number of patients evaluable for p16 CTC response may have been specimen integrity; our recent analyses have demonstrated that CTC specimens must be processed within 72 h of collection to yield a sufficient number of assessable CTCs, and several early specimens (31 out of the 496 collected) were not processed within this 72-h time frame.

These CTC results provide the evidence of the expected pharmacodynamic effects for this regimen in restoring tumor suppressor gene expression, and are consistent with the demonstrated increases in p16 expression and/or demethylation detected in clinical studies of decitabine in patients with solid tumor malignancies [7, 55, 56]. Of note, a similar lack of association between drug-induced increases in demethylation/expression of tumor-associated genes and clinical response was observed in a meta-analysis of 9 clinical studies of demethylating agents in solid tumor malignancies [56]. However, it is possible that an association between CTC p16 expression and response to FdCyd + THU might be observed with a larger number of patients—particularly, urothelial patients—for whom this regimen had the greatest activity in this study and for which reduced p16 expression was shown to be associated with poor prognosis in a meta-analysis of 37 studies [6].

Our pilot analysis of p16 expression in vimentin-positive CTCs revealed that increases in the proportion of p16-expressing VIM^+^ CTCs occurred in just 6 of the 12 patients evaluable for VIM^+^ CTC p16 expression changes, while all 12 patients exhibited increases in the proportion of p16-expressing CK^+^ CTCs. Though small sample size is again a caveat, it appears that increases in the proportion of p16-expressing CTCs occurred slightly later (if at all) in VIM^+^ CTCs relative to their CK^+^ counterparts. This limited or latent FdCyd-induced increase in the proportion of p16-expressing VIM^+^ CTCs, if verified by larger studies, may have biological and clinical implications given the importance of mesenchymal-associated characteristics such as enhanced motility and invasion in metastatic disease, as well as the apparent prognostic value of mesenchymal CTCs suggested by the recent studies [24–27]. Future preclinical studies exploring the molecular basis for this potential resistance to FdCyd-induced p16 expression in mesenchymal- and mixed E/M-phenotype tumor cells will be valuable in determining potential combination regimens, as well as highlighting specific patient populations that may benefit from FdCyd-based therapies. Finally, assessment of FdCyd-induced p16 expression changes in both CTCs and tumor biopsy specimens will be of great value in determining the adequacy of CTCs as a surrogate for tumor p16 expression, and this analysis is being performed in our ongoing phase 1 study of oral FdCyd + THU.

## Electronic supplementary material

Below is the link to the electronic supplementary material.

Supplementary file1 (DOCX 2323 kb)
