# Supplementary material for: Intravenous 5-fluoro-2′-deoxycytidine administered with tetrahydrouridine increases the proportion of p16-expressing circulating tumor cells in patients with advanced solid tumors
Source: Cancer Chemother Pharmacol. 2020 Apr 20;85(5):979–93. doi: 10.1007/s00280-020-04073-5 (PMC7188725; doi:10.1007/s00280-020-04073-5)
Supplement: Supplementary file 1 — Supplementary file1 (DOCX 2323 kb) [file 280_2020_4073_MOESM1_ESM.docx]

# Supplementary Methods and Results

## Statistical Considerations

Target progression-free survival (PFS) and objective response rate (ORR) values were selected based on historical benchmarks for the histologies in this trial. A retrospective review of 578 patients with metastatic breast cancer reported median time-to-treatment-failure (TTF) values of 2–4 months [[1](#_ENREF_1)], and another retrospective study of 140 patients found TTF values of 1–4 months and ORR values of 0–15% for breast cancer patients receiving 1 or more lines of salvage therapy following failure of first-line anthracycline-containing chemotherapy [[2](#_ENREF_2)]. For H&N squamous cell carcinoma, a retrospective analysis of 151 patients refractory to platinum-based chemotherapy treated with second-line therapies showed an overall survival of approximately 3.5 months and an ORR of 2.6% [[3](#_ENREF_3)], while a Phase 3 study of patients with recurrent/metastatic H&N cancer randomly assigned to receive cisplatin with cetuximab or with placebo found a statistically insignificant difference in median PFS between the groups: 2.7 months for patients on placebo and 4.2 months for patients on cisplatin and cetuximab [[4](#_ENREF_4)]. In the NSCLC setting, median PFS for treated patients with a poor performance status has been reported as 1 to 4 months, with ORR values of 9–19%, depending on the treatment [[5-9](#_ENREF_5)]. Similarly poor survival has been documented for patients with advanced urothelial transitional cell carcinoma, with median PFS values of 2.2–4.9 months and ORR of 6–60% in phase 2 salvage chemotherapy trials [[10](#_ENREF_10)].

The primary objective of this trial was to determine if the FdCyd + THU combination is associated with adequate ORR or PFS in patients with advanced NSCLC or urothelial, breast, or H&N carcinomas. These patients were previously treated with multiple prior chemotherapeutic regimens, so any reasonable evidence of benefit was interpreted as a positive indicator for the potential use of these agents in a subsequent, more definitive study. The study design was intended to discriminate between response rates of 20% versus 5% or 4-month (or, for the breast stratum, 6-month) PFS probabilities of 50% vs. 25% (corresponding to median PFS of 4 vs. 2 months, or, for the breast cancer stratum, 6 vs. 3 months). Given the historical data for these patient populations (as outlined above), FdCyd combined with THU was designated as worthy of further testing in NSCLC, urothelial, or H&N cancer if ≥ 6 objective responses (≥ 13%), or ≥ 18 instances of 4-month PFS (≥ 40%), were observed among 45 enrolled patients; for the breast stratum, the regimen was considered worthy of further testing if ≥ 5 objective responses (≥ 14%), or ≥ 15 instances of 6-month PFS (≥ 43%), were observed among 35 enrolled patients. As shown below, this design yielded adequate power (**≥** 84–90%) to detect a true ORR of at least 20% and to detect a 4- or 6-month PFS rate of at least 50% for each stratum. The design also yielded sufficiently high probabilities (**≥** .95–.96) of obtaining a negative result if the true ORR was ≤ 5% and the true 4- or 6-month PFS rate was ≤ 25%. In addition, the over-all probability of negative results across all 4 strata, under the over-all null hypothesis, was .85.

|  | **Power to detect true ORR of ≥20%** | **Power to detect true 4/6-month PFS rate of ≥50%** | **Probability of negative result if true ORR ≤5% and true 4/6-month PFS rate ≤25%** |
| --- | --- | --- | --- |
| **NSCLC** | **≥** 87% | **≥** 90% | **≥** .96 |
| **Breast** | **≥** 85% | **≥** 84% | **≥** .95 |
| **Urothelial** | **≥** 87% | **≥** 90% | **≥** .96 |
| **H&N** | **≥** 87% | **≥** 90% | **≥** .96 |

Probabilities were calculated assuming that ORR and PFS are not correlated; if they are positively correlated, as is likely, the probabilities would be higher than those shown.

## Preclinical validation of p16 expression as a PD biomarker for FdCyd + THU

*Cell culture*

EJ6 cells were obtained from the American Type Culture Collection (ATCC, Manassas, VA) and cultured in Dulbecco's Modified Eagle's medium (DMEM). OVCAR4 cells were obtained from the NCI Developmental Therapeutics Program Tumor Repository and cultured in Roswell Park Memorial Institute (RPMI) 1640 medium. All culture media included 10% fetal bovine serum, 100 I.U./mL penicillin, and 100 µg/mL streptomycin (penicillin-streptomycin from Lonza, Walkersville, MD). Cells were seeded at a concentration of 1×10^6^ cells in T25 flasks and incubated at 37°C in 5% CO_2_; cells were passaged every 7 days. Tetrahydrouridine (THU; NSC 112907) and 5-fluoro-2’-deoxycytidine (FdCyd; NSC 48006) were obtained from the NCI Division of Cancer Treatment and Diagnosis repository, while 2-deoxythymidine (dThy) was purchased from Affymetrix (Santa Clara, CA). Study drugs (10 μM FdCyd with 1 mM THU and 10 µM dThy) were added 24 hours after seeding and then re-applied once per passage, 4 days after each passaging. Cells were harvested after 0, 1, 2, 3, or 4 weeks of treatment. Cell counts were performed using a Cellometer Auto T4 (Nexcelom, Lawrence, MA).

*Western blot*

We tested several antibodies by Western blot, with the criterion for acceptance as an analytical reagent for CellSearch® assays being the presence of a single band at the appropriate molecular weight on a Western blot. The following monoclonal antibodies were purchased from Abcam (Cambridge, United Kingdom) for use in Western blot: anti-CDKN2A/p16 (clone EP435Y-129R, catalog number ab81278), anti-DNMT1 (clone EPR3521, catalog number ab134148), and GAPDH (clone EPR6256, catalog number ab128915). The LI-COR Biosciences (Lincoln, NE) IRDye® 800CW Goat anti-Rabbit IgG (H+L) secondary antibody (catalog number 926-32211) was used for detection. Cell lysates were prepared in Cell Extraction Buffer (Invitrogen/Thermo Fisher Scientific, Waltham, MA) containing cOmplete Protease Inhibitor Cocktail (Roche) and phenylmethanesulfonyl fluoride (Sigma-Aldrich, St. Louis, MO), and protein concentrations were determined using the Pierce BCA Protein Assay Kit (Thermo Fisher Scientific). Cell lysates were analyzed by SDS-PAGE (50 μg of total protein per lane) using NuPAGE 4-12% Bis-Tris gels (Invitrogen) and Precision Plus Protein molecular weight marker (Bio-Rad, Hercules, CA). Gels were run in 1X NuPAGE MOPS SDS running buffer (Invitrogen) and transferred using the Trans-Blot Turbo Transfer System (Bio-Rad). Membranes were blocked using Odyssey Blocking Buffer in PBS (LI-COR Biosciences, Lincoln, NE) and then incubated with the primary antibodies listed above, followed by probing with IRDye 800CW Streptavidin (LI-COR) diluted to 0.5 or 0.07 μg/mL for detection of p16 or DNMT1, respectively. Blots were scanned using an Odyssey IR Imager (LI‑COR).

*Immunofluorescence microscopy*

The AF 488−conjugated anti-CDKN2A/p16 EP435Y-129R antibody was used for immunofluorescence microscopy analysis of FdCyd/THU-treated EJ6 cells. Untreated or treated EJ6 cells or untreated OVCAR-4 cells were deposited on Marienfeld slides (Azer Scientific, Morgantown, PA), fixed with 10% neutral-buffered formalin (Sigma-Aldrich), and stored at −80°C until staining. Cells were stained with AF 488−conjugated EP435Y-129R and counterstained with DAPI. Sixty images of each treatment group were acquired at 20X magnification using a Nikon Eclipse Ni microscope (Melville, NY) equipped with a solid-state light source (Lumencor, Beaverton, OR) and Electron Multiplying Charged Coupled Device (EMCCD) camera (Andor, Belfast, UK). Images were analyzed by Definiens Developer XD (Cambridge, MA) using a custom rule set to detect cells by morphology and presence of DAPI and to determine both the average p16 signal within each cell as well as the average background within the image frame. In a separate experiment, FdCyd/THU-treated or untreated EJ6 cells were spiked into healthy donor blood (100 cells per mL blood), and sample processing and image analysis were performed using the 5-channel CellSearch® platform (Menarini Silicon Biosystems, Bryn Athyn, PA); the CellSearch® kit includes a staining reagent consisting of phycoerythrin (PE)-conjugated mouse monoclonal antibodies specific to cytokeratins and allophycocyanin (APC)-conjugated mouse anti-CD45 monoclonal antibody. Thresholding was performed using Spotfire (PerkinElmer, Waltham, MA) to determine the percentage of p16-positive cells within each treatment group. Information regarding the quantitation limit, accuracy, precision, and reproducibility of the CellSearch® system can be found in the CellSearch® package insert.

*Results for preclinical validation of a CellSearch*® *assay for CTC p16 expression*

Prior to pharmacodynamic analyses of clinical CTC specimens, we developed, validated, and established preclinical fitness-for-purpose for a CellSearch® assay to quantitate the proportion of p16-expressing tumor cells before and after treatment with the FdCyd + THU combination. To this end, we first assessed the ability of an Alexa Flor 488 (AF 488)−conjugated EP435Y-129R anti-p16 antibody to detect p16 expression following treatment with the combination of 10 μM FdCyd and 100 mM THU in EJ6 bladder carcinoma cells, which exhibit constitutive baseline *p16* silencing [[11](#_ENREF_11)]. Levels of p16 detectable by Western blot emerged after approximately 2 weeks of treatment and continued during weeks 3 and 4 (**Supplementary Fig. S1A**); this increase in p16 expression was coincident with reductions in DNMT1 expression, which was observed after 1 week of treatment and continued to decrease during weeks 2-4, consistent with the mechanism of action for a DNMT inhibitor. Increased p16 expression in EJ6 cells was also observed by immunofluorescence microscopy, wherein a small proportion of p16-expressing cells was observed starting at 1 week of treatment, and the proportion of p16-positive cells grew substantially by week 4 (**Supplementary Fig. S1B-C**). As expected, p16 expression was localized primarily in the nucleus, and control untreated OVCAR4 cells, which are known to express p16 [[12](#_ENREF_12)], exhibited substantial nuclear-localized p16 expression (**Supplementary Fig. S1B**).

We then applied the AF488-conjugated anti-CDKN2A/p16 EP435Y-129R antibody toward development of a clinically suitable assay for enumeration of p16-positive tumor cells before and after FdCyd + THU treatment. Using the CellSearch® platform, EpCAM/CD146-positive EJ6 cells spiked into human blood specimens were isolated and analyzed via immunofluorescence microscopy to enumerate as tumor cells those that are nucleated (i.e., stained by DAPI), expressing the epithelial marker cytokeratin and the tumor marker MUC1 [[13](#_ENREF_13), [14](#_ENREF_14)], and negative for the leukocyte marker CD45 (the latter three detected by fluorescence-conjugated antibodies to the respective proteins)—and to also enumerate p16-positive tumor cells. Following EpCAM/CD146 purification, blood specimens spiked with untreated EJ6 cells yielded many CD45^−^/cytokeratin^+^/MUC1^+^/DAPI^+^ cells, but these cells were largely negative for p16 expression (**Supplementary Fig. S1D-E**). In contrast, blood specimens spiked with FdCyd + THU–treated EJ6 cells contained many CD45^−^/cytokeratin^+^/MUC1^+^/DAPI^+^ cells that were also positive for p16 expression according to the CellSearch® algorithm (**Supplementary Fig. S1D-E**). The pattern of p16 expression in these cells was largely pan-nuclear, confirming the suitability of this marker for CellSearch® analysis. Though the tumor marker MUC1 is a membrane-anchored protein, we also observe MUC1 in the nuclei and cytoplasm of some CTCs, consistent with previous reports [[15](#_ENREF_15), [16](#_ENREF_16)]. Together, these data demonstrate that use of the AF488-conjugated EP435Y-129R antibody on the CellSearch® platform enables clinically suitable identification and enumeration of p16-positive tumor cells in human blood specimens.

## Pharmacokinetics

### *Pharmacokinetics Methods*

Blood samples for PK analysis were obtained from 18 patients (9 from the urothelial stratum and 3 from all other strata) at the following time points during cycle 1: day 1 pre-treatment; day 1 at 15 minutes, 30 minutes, and 1, 2, and 2.5 hours during the infusion; day 1 at 15 minutes, 30 minutes, and 1, 2, 4, and 6 hours post completion of infusion; and on days 2, 3, 4, and 5 prior to treatment. Each 3-mL blood sample was collected in a heparinized tube preloaded with 30 μL of 100 mg/mL of zebularine to minimize *ex vivo* deamination of FdCyd during processing and storage. Samples were gently inverted 3 times, placed on ice, and then centrifuged at 600 × g for 10 minutes at 4°C. The resulting plasma samples were then frozen and stored at or below ‑20°C prior to analysis. Plasma levels of FdCyd, FdUrd, and FU were determined with a previously described LC-MS/MS assay [[17](#_ENREF_17)]. THU was quantified in plasma using a separate LC-MS/MS assay [[18](#_ENREF_18), [19](#_ENREF_19)]. Concentration-time data were analyzed non-compartmentally using PK Solutions (Summit Research Services, Montrose, CO).

Urine samples for PK analysis were collected at every void from 0 to 24-hours post-drug infusion on cycle 1 day 1. Each sample was centrifuged at 600 × g for 1 minute at 4°C to sediment debris, and then 0.5 mL was transferred into each of 3 cryogenic vials containing 5 μL of 100 mg/mL zebularine each. Vials were then frozen and stored at or below ‑20°C prior to LC‑MS/MS analysis.

### *Pharmacokinetics Results*

Mean plasma pharmacokinetic parameters were generated for FdCyd, FdUrd, 5-fluorouracil (FU), and THU based on data from 18 patients (9 from the urothelial stratum and 3 each from all other strata; **Supplementary Table S2**). Mean plasma concentration × time plots for each analyte are shown in **Supplementary Fig. S3A**. For this phase 2 dose of FdCyd + THU, the mean C_max_ of FdCyd was 3296 (± 1297) ng/mL, and the AUC_0-∞_ was 766 (± 253) µg*min/mL; no significant differences in FdCyd C_max_ or AUC_0-∞_ values were found among the 4 strata (according to unpaired t-tests with Welch’s correction; **Supplementary Fig. S3B**). Lower levels of the primary metabolites, FdUrd and FU, appeared in plasma, with a FdCyd/FdUrd ratio for C_max_ (mean and standard deviation of individual ratios) of 162 (± 48) and for AUC_0-∞_ of 97.6 (± 28.1). The corresponding ratios for FdCyd/FU were 383 (± 236) and 267 (± 197). All of these values are similar to those reported for IV administration of the same doses in the prior phase 1 study [[20](#_ENREF_20)]. The lower clearance and longer half-life of THU is similar to prior observations and consistent with its relative resistance to metabolism [[20](#_ENREF_20)].

Analysis of Day 1, 24-hour urine collections demonstrated minimal urinary excretion of FdCyd, FdUrd, or FU (0-3% of dose), consistent with further downstream metabolism of FU. Urinary excretion of THU was highly variable (1–100% of dose).

# Supplementary Tables

## Supplementary Table S1. Grade ≥ 2 treatment-related adverse events.

| **Adverse event** | **Grade 2** | **Grade 3** | **Grade 4** | **Grade 5** |
| --- | --- | --- | --- | --- |
| Hematological |  |  |  |  |
| Anemia | 25 | 9 | — | — |
| Leukopenia | 13 | 11 | 4 | — |
| Lymphopenia | 15 | 28 | 9 | — |
| Neutropenia | 10 | 8 | 4 | — |
| Febrile neutropenia | — | 1 | 1 | — |
| Thrombocytopenia | 5 | 5 | — | — |
| Gastrointestinal |  |  |  |  |
| Abdominal distension | — | 1 | — | — |
| Abdominal pain | 7 | 3 | — | — |
| Anorexia | 8 | 2 | — | — |
| Colitis | — | 1 | — | — |
| Constipation | 1 | 1 | — | — |
| Diarrhea | 5 | 7 | — | — |
| Duodenal ulcer | 1 | — | — | — |
| Dysphagia | — | 1 | — | — |
| Esophagitis | — | 1 | — | — |
| Gastritis | 1 | — | — | — |
| Ileus | — | 1 | — | — |
| Mucositis oral | 2 | 3 | — | — |
| Nausea | 4 | 3 | — | — |
| Vomiting | 5 | 4 | — | — |
| Electrolyte |  |  |  | — |
| Dehydration | 1 | 4 | — | — |
| Hyperkalemia | 1 | — | — |  |
| Hypoalbuminemia | 18 | — | — | — |
| Hypocalcemia | 4 | — | — | — |
| Hypokalemia | — | 1 | — | — |
| Hypomagnesemia | 1 | — | — | — |
| Hyponatremia | 1 | 5 | 1 | — |
| Hypophosphatemia | 10 | 5 | — | — |
| Cardiac and vascular |  |  |  |  |
| Cardiac arrest | — | — | — | 1 |
| Hypertension | 2 | 1 | — | — |
| Sinus tachycardia | 1 | 1 | — | — |
| Superficial thrombophlebitis | 1 | — | — | — |
| Infection |  |  |  |  |
| Enterocolitis infectious | 1 | — | — | — |
| Herpes | 1 | — | — | — |
| Mucosal infection | 1 | 1 | — | — |
| Skin |  |  |  |  |
| Palmar-plantar erythrodysesthesia syndrome | 2 | — | — | — |
| Rash maculo-papular | 2 | 2 | — | — |
| Skin hypopigmentation | 1 | — | — | — |
| Investigations |  |  |  |  |
| Alanine aminotransferase increased | 1 | 1 | — | — |
| Alkaline phosphatase increased | 1 | — | — | — |
| Aspartate aminotransferase increased | 2 | 1 | — | — |
| Blood bilirubin increased | — | 1 | — | — |
| Creatinine increased | — | 1 | — | — |
| Hemoglobin increased | 1 | — | — | — |
| Weight loss | 3 | 1 | — | — |
| Musculoskeletal |  |  |  |  |
| Generalized muscle weakness | 1 | — | — | — |
| Muscle weakness lower limb | 1 | — | — | — |
| Myalgia | 1 | — | — | — |
| Psychiatric and nervous system |  |  |  |  |
| Agitation | — | — | 1 | — |
| Anxiety | — | 1 | — | — |
| Peripheral sensory neuropathy | 1 | — | — | — |
| Other |  |  |  |  |
| Acute kidney injury | — | 1 | — | — |
| Dyspnea | 1 | — | 1 | — |
| Hiccups | 1 | — | — | — |
| Edema limbs | 3 | — | — | — |
| Fatigue | 23 | 9 | — | — |
| Fever | 4 | — | — | — |
| Infusion site extravasation | 2 | — | — | — |
| Pain | 1 | — | — | — |

All ≥ grade 2 adverse events that are at least possibly attributed to FdCyd and/or THU are shown.

## Supplementary Table S2. Non-compartmental plasma pharmacokinetic parameters for FdCyd, FdUrd, FU, and THU.

| **Compound** | **C_max_**  (μg/mL) | **t_max_**  (min) | **t_1/2_**  (min) | **AUC_0-t_**  (μg*min/mL) | **AUC_0-inf_**  (μg*min/mL) | **Vd**  (L/m^2^) | **Vss**  (L/m^2^) | **Cl**  (mL/min/m^2^) |
| --- | --- | --- | --- | --- | --- | --- | --- | --- |
| **FdCyd** | 3296 (1297) | 162 (24) | 63 (18) | 766 (253) | 780 (259) | 12.2 (3.4) | 28.8 (8.2) | 142 (46) |
| **FdUrd** | 20.9 (6.1) | 157 (39) | 191 (107) | 6.49 (1.97) | 8.26 (2.59) | – | – | – |
| **FU** | 11.1 (5.1) | 201 (96) | 169 (79) | 3.62 (2.30) | 4.40 (2.60) | – | – | – |
| **THU** | 17408 (2395) | 154 (21) | 452 (135) | 8425 (2552) | 9528 (3669) | 26.3 (5.2) | 20.8 (2.8) | 43.2 (14.1) |

Mean (standard deviation) values are shown for each parameter (*n*= 18 patients).

# Supplementary Figures


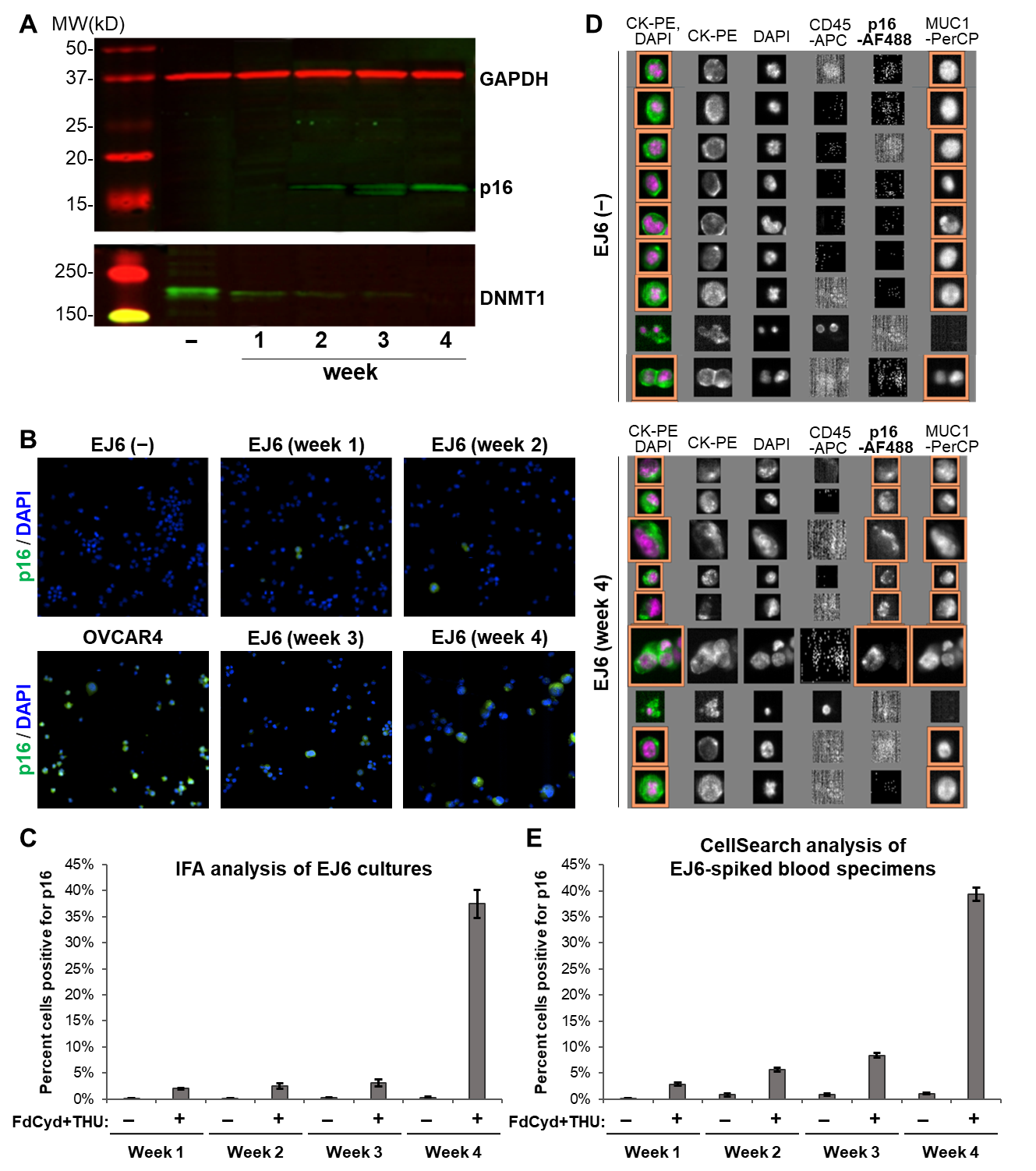


## Supplementary Figure S1. Preclinical validation of a CellSearch® assay for enumeration of p16-positive CK^+^ tumor cells following exposure to FdCyd + THU. A, Treatment of EJ6 cells with the combination of FdCyd (10 μM) and THU (100 mM) induces increased p16 protein expression and decreased DNMT1 expression. Protein levels were detected by Western blot in untreated cells (−) or at the indicated time points following treatment. B, Immunofluorescence microscopy images of untreated (−) and FdCyd + THU−treated EJ6 cells at the indicated time points or untreated OVCAR4 cells, which are known to express appreciable levels of p16 (8); cells were stained with DAPI (blue) and an AF 488−conjugated antibody to p16 (green). C, Quantitation of the percentage of cells positive for p16 by IFA analysis of untreated or FdCyd + THU−treated EJ6 cells. Average values are shown for each time point and treatment (*n* = 3 slides for each); error bars represent standard deviation. D, CellSearch® analysis of untreated (top) and FdCyd + THU−treated (bottom) EJ6 cells spiked into healthy donor blood demonstrates treatment-induced upregulation of p16 positivity. Treated EJ6 cells were incubated for 4 weeks with 10 μM FdCyd and 100 mM THU. EJ6-spiked blood samples were analyzed using a 5-channel CellSearch® platform; tumor cells were identified as those positive for cytokeratin (CK), DAPI, and MUC1 and negative for CD45 (a marker of antigen-presenting cells, APC). Antibodies used for CellSearch® analysis include AF488-conjugated anti-p16 (“p16‑AF488”), PE-conjugated anti-CK (“CK‑PE”), APC-conjugated anti-CD45 (“CD45-APC”), and PerCP-conjugated MUC1 (“MUC1‑PerCP”). Orange boxes indicate cells identified as positive for the given marker by CellSearch® analysis algorithms. E, Quantitation of the percentage of tumor cells positive for p16 by CellSearch® analysis of blood specimens spiked with either untreated or FdCyd + THU−treated EJ6 cells. Average values are shown for each time point and treatment (*n* = 3 specimens for each); error bars represent standard deviation.


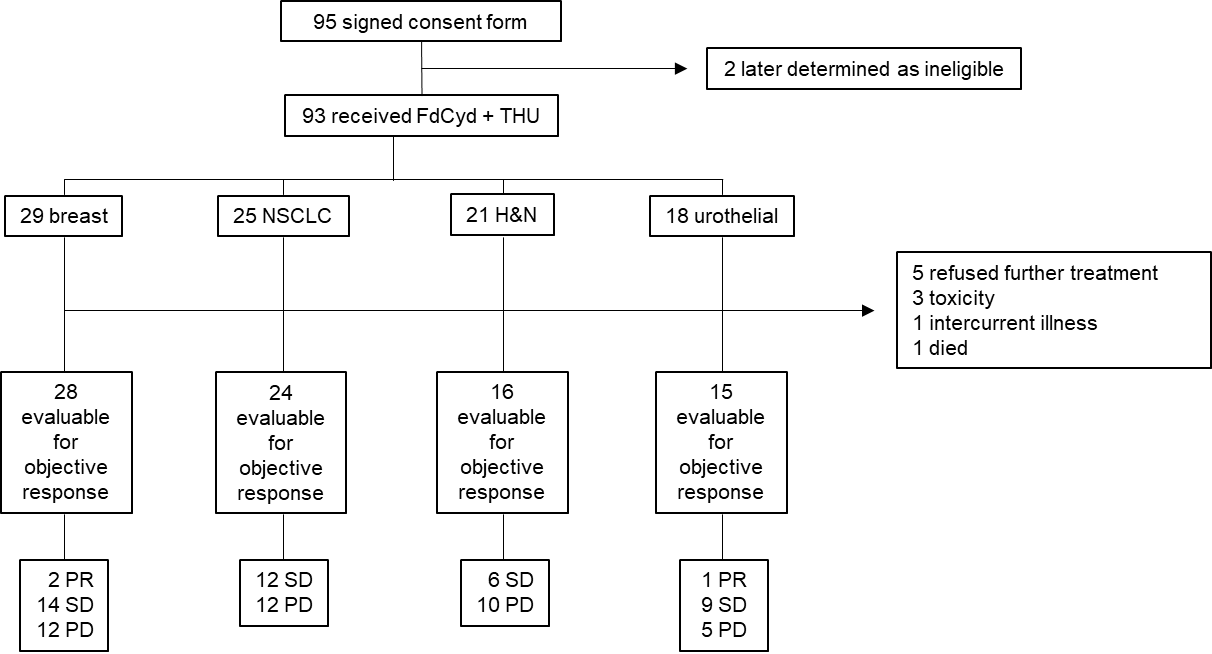


## **Supplementary Figure S2.** CONSORT (Consolidated Standards of Reporting Trials) diagram.


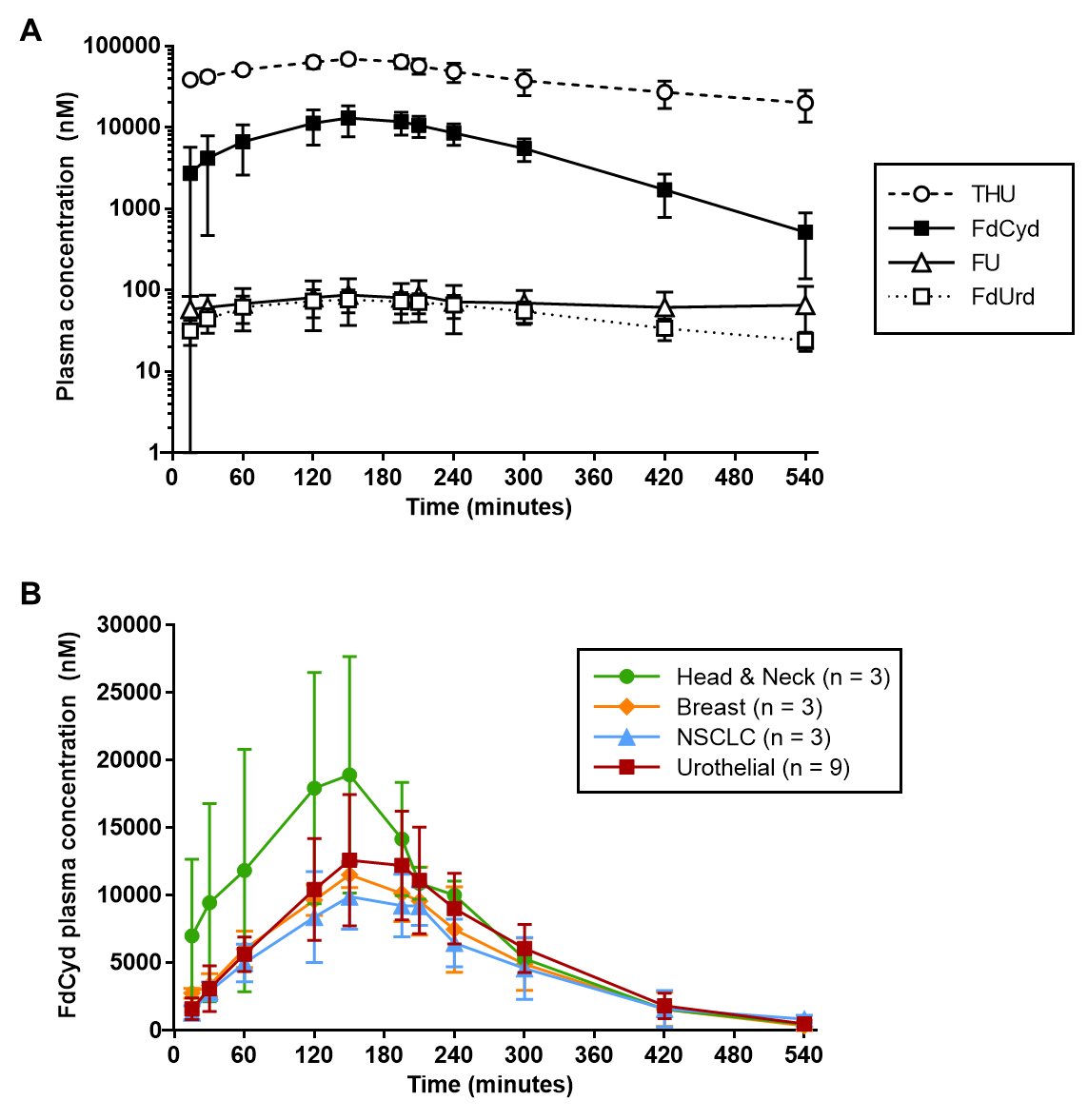


## Supplementary Figure S3. Plasma concentrations of THU, FdCyd, FU, and FdUrd after single-dose intravenous administration of FdCyd and THU. A, Mean plasma concentrations of THU, FdCyd, and FdCyd metabolites (*n* = 18 patients; error bars indicate standard deviation). B, Mean FdCyd plasma concentrations by stratum: head & neck (green circles; *n* = 3 patients), breast (orange diamonds; *n* = 3), NSCLC (blue triangles; *n* = 3), and urothelial (red squares; *n* = 9). Error bars indicate standard deviation.


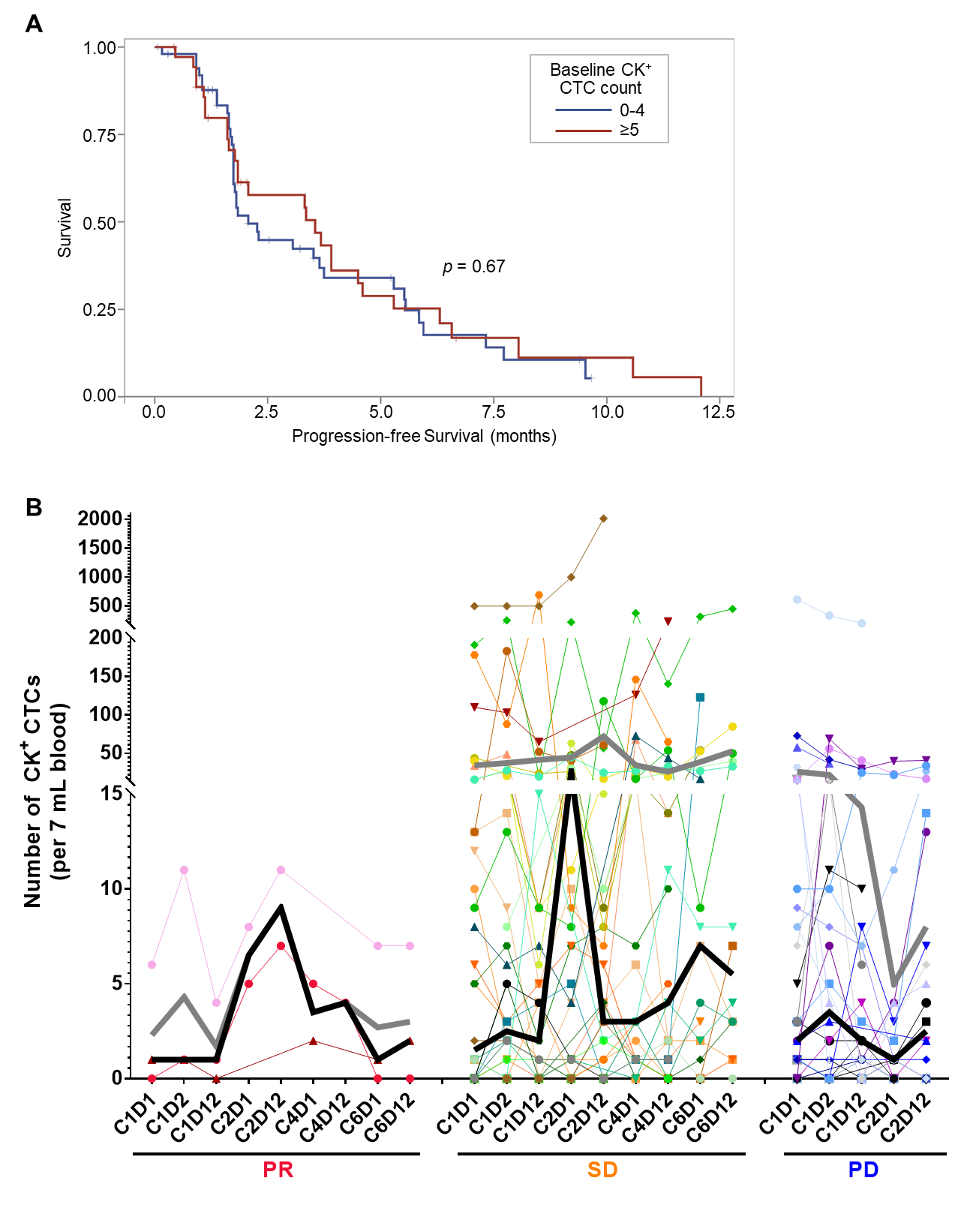


## Supplementary Figure S4. CK^+^ CTC counts are not associated with response to FdCyd + THU. A, Pre-treatment (cycle 1 day 1) cytokeratin^+^ CTC count is not predictive of progression-free survival (PFS) in response to FdCyd + THU. Kaplan-Meier curves for patients with pre-treatment CK^+^ CTC counts of 0-4 (blue) or ≥ 5 (red) and the *p-*value from a log-rank test comparing these two groups are shown. A cut-off of 5 CTCs for high vs. low baseline CTC count was selected based on prior studies [[21-23](#_ENREF_21)], though use of 0, 3, or 4 CTCs as a cut-off (as has also been employed in various previous studies [[22](#_ENREF_22), [24-26](#_ENREF_24)]) did not change the outcome of this analysis; in each case, there was no significant difference in PFS between the pre-treatment CK^+^ CTC-high and -low groups according to log-rank tests (*p* = 0.67-0.96). Data are shown for all patients who provided pre-treatment CTC specimens (*n* = 87); vertical lines represent censoring events. B, Post-treatment changes in CTC number, grouped by best response to therapy. PR, partial response; SD, stable disease; PD, progressive disease. Thick black lines indicate median CTC counts at each time point, while thick grey lines indicate mean CTC counts. Data are shown for all patients evaluable for objective response who also provided blood specimens for CTC analysis (*n* = 76)*.*


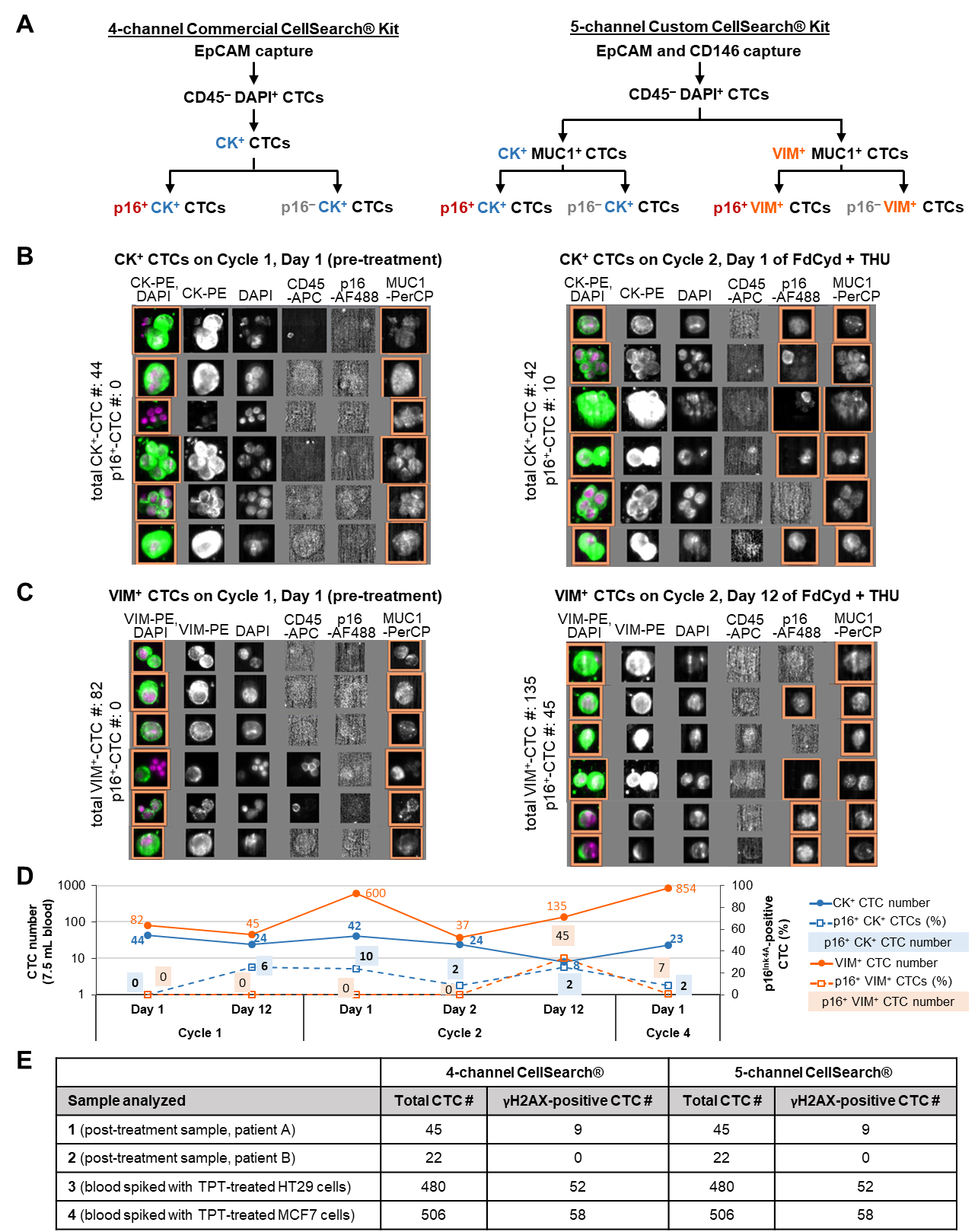


## Supplementary Figure S5. Implementation and validation of the 5-channel CellSearch® system for assessing p16 expression in CK^+^ and VIM^+^ CTCs. A, Schematic for CTC p16 analysis using the 4-channel commercial CellSearch® kit (left) and our laboratory-developed novel 5-channel custom CellSearch® kit (right). The anti-vimentin antibody used in the 5-channel assay (clone V9, Santa Cruz Biotechnology, Dallas, TX) was previously validated for use in identifying CTCs of non-epithelial origin [[27](#_ENREF_27)]. B and C, 5-channel CellSearch® analysis of CK^+^ (B) and VIM^+^ (C) pre- (left) and post-treatment (right) CTCs from a patient treated with FdCyd + THU, showing a treatment-induced increase in the number of p16^+^CK^+^ and p16^+^VIM^+^ CTCs. D, Longitudinal 5-channel CellSearch® evaluation of CK^+^ and VIM^+^ CTC enumeration and p16 positivity for specimens from the FdCyd + THU–treated patient shown in A and B. For CK^+^ (blue) and VIM^+^ (orange) CTCs, the CTC number (left axis; solid lines) and percentage of CTCs positive for p16 (right axis, dashed lines) are shown for the indicated time points. E, Comparison of 4- vs. 5-channel CellSearch® analysis of CTC enumeration and γH2AX positivity following treatment with DNA damage–inducing agents. Samples 1 and 2 were collected from 2 different patients enrolled on a trial of a DNA damage–inducing investigational combination therapy, while samples 3 and 4 consisted of healthy donor blood spiked with either HT29 or MCF7 cells that had been treated with 1 μM topotecan for 2 hours. The 4- and 5-channel CellSearch® platforms produced identical results for these 4 pilot samples.


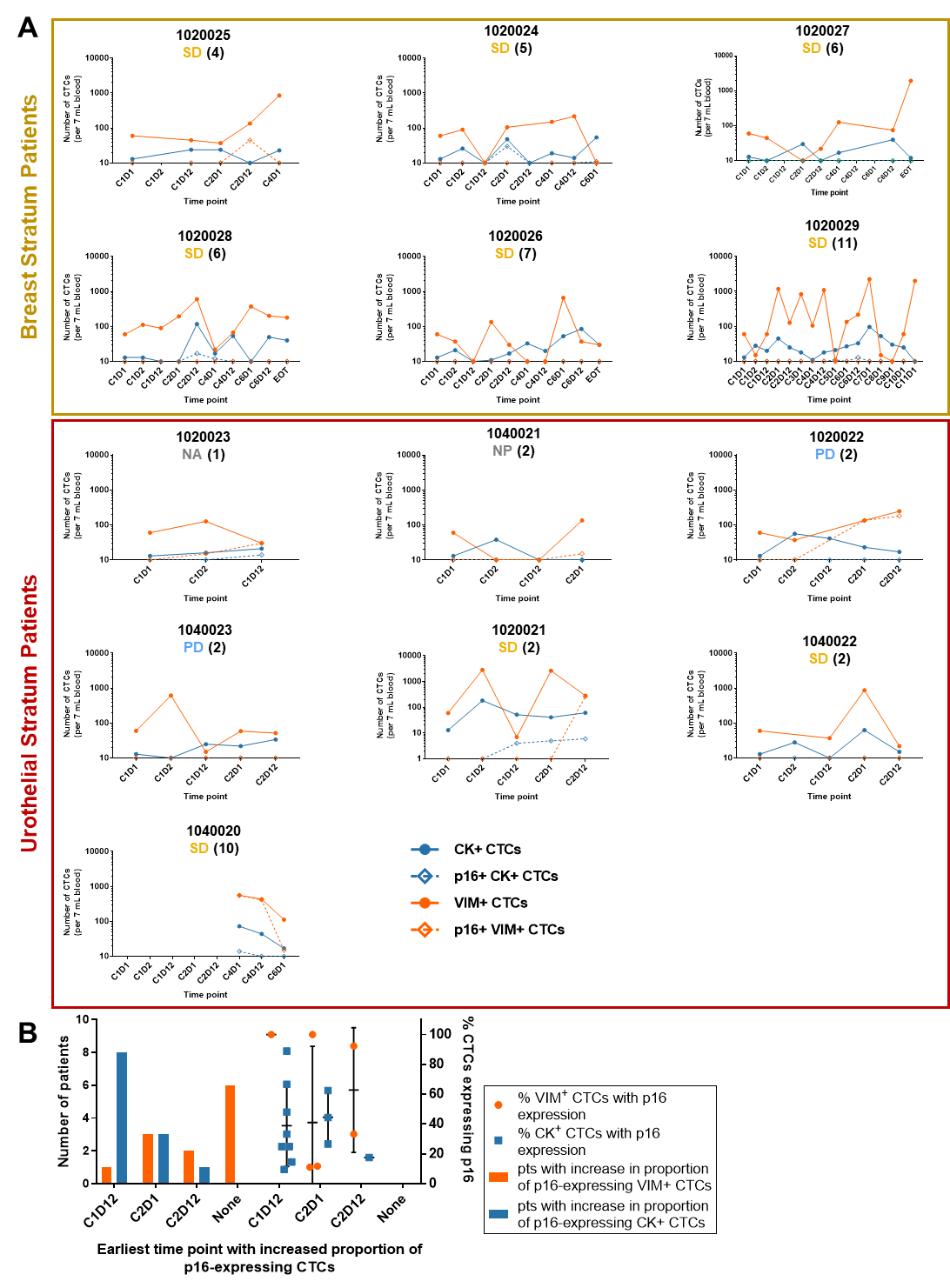


## Supplementary Figure S6. Cytokeratin-positive versus vimentin-positive CTC enumeration and p16 expression. A, For each patient, the numbers of cytokeratin-positive (CK^+^) and vimentin-positive (VIM^+^) CTCs per 7 mL blood at each time point are shown by solid blue and orange lines, respectively (left-hand y-axis), while the numbers of p16-positive cells within each CTC subpopulation (CK^+^ or VIM^+^) are shown by dashed blue and orange lines, respectively. Patients are grouped according to tumor type: breast (top, gold) or urothelial (bottom, red). No patients in the NSCLC or H&N strata were assessed for VIM^+^ CTCs. Best response to therapy (SD, stable disease; PD, progressive disease; NA, not assessed; NP, not applicable per protocol) and number of treatment cycles completed are noted under each patient number. B, Histogram showing frequency (left) and magnitude (right) for the earliest time point at which an increase in the proportion of p16-expressing VIM^+^ (orange) or CK^+^ (blue) CTCs was observed.

# Supplementary References

1. Tacca O, LeHeurteur M, Durando X, Mouret-Reynier M-A, Abrial C et al (2009) Metastatic Breast Cancer: Overall Survival Related to Successive Chemotherapies. What Do We Gain After the Third Line? Cancer Invest 27(1): 81 - 85.

2. Campora E, Gardin G, Gasco M, Rosso R, Santi L (1999) Metastatic breast cancer patients failing first-line, anthracycline-containing chemotherapy: is further therapy of benefit? Anticancer Res 19(4c): 3429-32.

3. León X HR, Constenla M, Rocca A, Stupp R, Kovács AF, Amellal N, Bessa EH, Bourhis J (2005) A retrospective analysis of the outcome of patients with recurrent and/or metastatic squamous cell carcinoma of the head and neck refractory to a platinum-based chemotherapy. Clin Oncol (R Coll Radiol) 17(6): 418-24.

4. Burtness B, Goldwasser MA, Flood W, Mattar B, Forastiere AA (2005) Phase III Randomized Trial of Cisplatin Plus Placebo Compared With Cisplatin Plus Cetuximab in Metastatic/Recurrent Head and Neck Cancer: An Eastern Cooperative Oncology Group Study*.* J Clin Oncol 23(34): 8646-8654. <https://doi.org/10.1200/jco.2005.02.4646>

5. Goss G, Ferry D, Wierzbicki R, Laurie SA, Thompson J et al (2009) Randomized Phase II Study of Gefitinib Compared With Placebo in Chemotherapy-Naive Patients With Advanced Non-Small-Cell Lung Cancer and Poor Performance Status*.* J Clin Oncol: JCO.2008.18.4408. <https://doi.org/10.1200/jco.2008.18.4408>

6. Adjei AA, Mandrekar SJ, Dy GK, Molina JR, Adjei AA et al (2010) Phase II trial of pemetrexed plus bevacizumab for second-line therapy of patients with advanced non-small-cell lung cancer: NCCTG and SWOG study N0426*.* J Clin Oncol 28(4): 614-9. <https://doi.org/10.1200/jco.2009.23.6406>

7. Fukuoka M, Yano S, Giaccone G, Tamura T, Nakagawa K et al (2003) Multi-Institutional Randomized Phase II Trial of Gefitinib for Previously Treated Patients With Advanced Non-Small-Cell Lung Cancer*.* J Clin Oncol 21(12): 2237-2246. <https://doi.org/10.1200/jco.2003.10.038>

8. Herbst RS, O'Neill VJ, Fehrenbacher L, Belani CP, Bonomi PD et al (2007) Phase II Study of Efficacy and Safety of Bevacizumab in Combination With Chemotherapy or Erlotinib Compared With Chemotherapy Alone for Treatment of Recurrent or Refractory Non Small-Cell Lung Cancer*.* J Clin Oncol 25(30): 4743-4750. <https://doi.org/10.1200/jco.2007.12.3026>

9. Shepherd FA, Rodrigues Pereira J, Ciuleanu T, Tan EH, Hirsh V et al (2005) Erlotinib in Previously Treated Non-Small-Cell Lung Cancer*.* N Engl J Med 353(2): 123-132. <https://doi.org/10.1056/NEJMoa050753>

10. Sonpavde G, Galsky MD, Hutson TE (2008) Current optimal chemotherapy for advanced urothelial cancer*.* Expert Rev Anticancer Ther 8(1): 51-61. <https://doi.org/10.1586/14737140.8.1.51>

11. Cheng JC, Matsen CB, Gonzales FA, Ye W, Greer S et al (2003) Inhibition of DNA methylation and reactivation of silenced genes by zebularine*.* J Natl Cancer Inst 95(5): 399-409.

12. Kubo A, Nakagawa K, Varma RK, Conrad NK, Cheng JQ et al (1999) The p16 status of tumor cell lines identifies small molecule inhibitors specific for cyclin-dependent kinase 4*.* Clin Cancer Res 5(12): 4279-4286.

13. Horm TM, Schroeder JA (2013) MUC1 and metastatic cancer: expression, function and therapeutic targeting*.* Cell Adh Migr 7(2): 187-98. <https://doi.org/10.4161/cam.23131>

14. Nath S, Mukherjee P (2014) MUC1: a multifaceted oncoprotein with a key role in cancer progression*.* Trends Mol Med 20(6): 332-42. <https://doi.org/10.1016/j.molmed.2014.02.007>

15. Ghosh SK, Pantazopoulos P, Medarova Z, Moore A (2013) Expression of Underglycosylated MUC1 Antigen in Cancerous and Adjacent Normal Breast Tissues*.* Clin Breast Cancer 13(2): 109-118. <https://doi.org/https://doi.org/10.1016/j.clbc.2012.09.016>

16. Kumar P, Lindberg L, Thirkill TL, Ji JW, Martsching L et al (2012) The MUC1 Extracellular Domain Subunit Is Found in Nuclear Speckles and Associates with Spliceosomes*.* PLOS ONE 7(8): e42712. <https://doi.org/10.1371/journal.pone.0042712>

17. Holleran JL, Eiseman JL, Parise RA, Kummar S, Beumer JH (2016) LC-MS/MS assay for the quantitation of FdCyd and its metabolites FdUrd and FU in human plasma*.* J Pharm Biomed Anal 129: 359-366. <https://doi.org/10.1016/j.jpba.2016.07.027>

18. Parise RA, Egorin MJ, Eiseman JL, Joseph E, Covey JM et al (2007) Quantitative determination of the cytidine deaminase inhibitor tetrahydrouridine (THU) in mouse plasma by liquid chromatography/electrospray ionization tandem mass spectrometry*.* Rapid Commun Mass Spectrom 21(13): 1991-7. <https://doi.org/10.1002/rcm.3054>

19. Beumer JH, Eiseman JL, Parise RA, Florian JA, Jr., Joseph E et al (2008) Plasma pharmacokinetics and oral bioavailability of 3,4,5,6-tetrahydrouridine, a cytidine deaminase inhibitor, in mice*.* Cancer Chemother Pharmacol 62(3): 457-64. <https://doi.org/10.1007/s00280-007-0625-2>

20. Holleran JL, Beumer JH, McCormick DL, Johnson WD, Newman EM et al (2015) Oral and intravenous pharmacokinetics of 5-fluoro-2'-deoxycytidine and THU in cynomolgus monkeys and humans*.* Cancer Chemother Pharmacol 76(4): 803-11. <https://doi.org/10.1007/s00280-015-2857-x>

21. Cristofanilli M, Budd GT, Ellis MJ, Stopeck A, Matera J et al (2004) Circulating tumor cells, disease progression, and survival in metastatic breast cancer*.* N Engl J Med 351(8): 781-91. <https://doi.org/10.1056/NEJMoa040766>

22. Munzone E, Botteri E, Sandri MT, Esposito A, Adamoli L et al (2012) Prognostic value of circulating tumor cells according to immunohistochemically defined molecular subtypes in advanced breast cancer*.* Clin Breast Cancer 12(5): 340-6. <https://doi.org/10.1016/j.clbc.2012.07.001>

23. Yang JD, Campion MB, Liu MC, Chaiteerakij R, Giama NH et al (2016) Circulating tumor cells are associated with poor overall survival in patients with cholangiocarcinoma*.* Hepatology 63(1): 148-58. <https://doi.org/10.1002/hep.27944>

24. Olmos D, Baird RD, Yap TA, Massard C, Pope L et al (2011) Baseline circulating tumor cell counts significantly enhance a prognostic score for patients participating in phase I oncology trials*.* Clin Cancer Res 17(15): 5188-96. <https://doi.org/10.1158/1078-0432.Ccr-10-3019>

25. Resel Folkersma L, San Jose Manso L, Galante Romo I, Moreno Sierra J, Olivier Gomez C (2012) Prognostic significance of circulating tumor cell count in patients with metastatic hormone-sensitive prostate cancer*.* Urology 80(6): 1328-32. <https://doi.org/10.1016/j.urology.2012.09.001>

26. Kaifi JT, Kunkel M, Dicker DT, Joude J, Allen JE et al (2015) Circulating tumor cell levels are elevated in colorectal cancer patients with high tumor burden in the liver*.* Cancer Biol Ther 16(5): 690-8. <https://doi.org/10.1080/15384047.2015.1026508>

27. Balasubramanian P, Kinders RJ, Kummar S, Gupta V, Hasegawa D et al (2017) Antibody-independent capture of circulating tumor cells of non-epithelial origin with the ApoStream(R) system*.* PLoS One 12(4): e0175414. <https://doi.org/10.1371/journal.pone.0175414>
